# Application of Metaheuristics for Optimizing Predictive Models in iHealth: A Case Study on Hypotension Prediction in Dialysis Patients

**DOI:** 10.3390/biomimetics10050314

**Published:** 2025-05-12

**Authors:** Felipe Cisternas-Caneo, María Santamera-Lastras, José Barrera-Garcia, Broderick Crawford, Ricardo Soto, Cristóbal Brante-Aguilera, Alberto Garcés-Jiménez, Diego Rodriguez-Puyol, José Manuel Gómez-Pulido

**Affiliations:** 1 Escuela de Ingeniería Informática, Pontificia Universidad Católica de Valparaíso, Avenida Brasil 2241, Valparaíso 2362807, Chile; felipe.cisternas.c@mail.pucv.cl (F.C.-C.); jose.barrera@pucv.cl (J.B.-G.); ricardo.soto@pucv.cl (R.S.); 2 Department of Medicine and Medical Specialties, Universidad de Alcalá, Alcalá de Henares, 28805 Madrid, Spain; maria.santamera@uah.es (M.S.-L.); diego.rodriguez@uah.es (D.R.-P.); 3 Health Computing and Intelligent Systems Research Group (HCIS), Universidad de Alcalá, Alcalá de Henares, 28805 Madrid, Spain; alberto.garces@uah.es (A.G.-J.); jose.gomez@uah.es (J.M.G.-P.); 4 Escuela de Negocios y Economía, Pontificia Universidad Católica de Valparaíso, Amunátegui 1838, Viña del Mar 2580129, Chile; 5 Escuela de Ingeniería en Construcción y Transporte, Pontificia Universidad Católica de Valparaíso, Avenida Brasil 2147, Valparaíso 2362804, Chile; cristobal.brante@pucv.cl; 6 Department of Computer Science, Universidad de Alcalá, Alcalá de Henares, 28805 Madrid, Spain; 7 Ramón y Cajal Institute for Health Research (IRYCIS), 28034 Madrid, Spain; 8 Nephrology Department and Research Foundation, Hospital Universitario Principe de Asturias, 28805 Madrid, Spain

**Keywords:** intradialytic hypotension, chronic kidney diseases, machine learning, metaheuristic, feature selection, optimization

## Abstract

Intradialytic hypotension (IDH) is a critical complication in patients with chronic kidney disease undergoing dialysis, affecting both patient safety and treatment efficacy. This study examines the application of advanced machine learning techniques, combined with metaheuristic optimization methods, to improve predictive models for intradialytic hypotension (IDH) in hemodialysis patients. Given the critical nature of IDH, which can lead to significant complications during dialysis, the development of effective predictive tools is vital for improving patient safety and outcomes. Dialysis session data from 758 patients collected between January 2016 and October 2019 were analyzed. Particle Swarm Optimization, Grey Wolf Optimizer, Pendulum Search Algorithm, and Whale Optimization Algorithm were employed to reduce the feature space, removing approximately 45% of clinical and analytical variables while maintaining high recall for the minority class of patients experiencing hypotension. Among the evaluated models, the XGBoost classifier showed superior performance, achieving a macro F-score of 0.745 with a recall of 0.756 and a precision of 0.718. These results highlight the effectiveness of the combined approach for early identification of patients at risk for IDH, minimizing false negatives, and improving clinical decision-making in nephrology.

## 1. Introduction

Dialysis and transplantation have enabled many patients with advanced chronic kidney disease (CKD) to remain alive and have a good quality of life for many years. Although early renal engraftment is the desirable treatment for these patients, this is not always possible, partly because of the limited availability of organs and partly because of patient characteristics. The prevalence of CKD in its most advanced stages, and specifically those requiring RRT, has grown by almost 30% in Spain in the last decade. According to data from the O.N.T./S.E.N. Registry, the number of people on RRT hemodialysis, peritoneal dialysis, or transplantation has already reached 1363 per million population, totaling 64,600 people as of 2020, as reported in [1]. Consequently, in 2014, 26,533 patients in Spain needed dialysis techniques to stay alive, and of these, 23,519 patients required a technique known as hemodialysis (HD). If we compare these figures with the prevalence of other diseases, they are not very significant. However, the impact on individuals and families is brutal, and the economic cost is disproportionate, as 2.5% of all Spanish healthcare expenditure is devoted to treating patients with end-stage CKD [2].

Significant advances in hemodialysis techniques in recent decades have allowed us to improve the management of intradialytic hypotension (IDH) as a recurrent problem in patients receiving hemodialysis (HD) treatment. Nevertheless, this phenomenon is still too frequent (5–40% of sessions) [3,4] and is associated with high patient morbidity and mortality and multiple complications. There are no guidelines or systematic treatments to manage this complication, and different maneuvers are used to control it [5]. IDH has three essential components: a drop of more than 20 mmHg in systolic blood pressure (SBP) or more than 10 mmHg in mean arterial pressure (MAP), the presence of symptoms due to ischemia of different organs, and interventions by dialysis staff [6]. Identifying the occurrence of HDI during HD treatment is not a straightforward task due to the numerous variables or factors involved, including the type of dialyzer, dialyzer temperature, patient type, dialysis modality, and medical criteria, among others. HDI involves complex factors, including circulating volume, fluid removal rate, the rate of “filling” the intravascular space, the osmolar gradient between compartments, and impairment of compensatory mechanisms due to underlying medical conditions, diseases, or medications. In addition, some patients are at higher risk; studies suggest that women, the elderly with isolated systolic hypertension, diabetics, and those with documented autonomic neuropathy or heart disease are at higher risk of IDH.

Thus, this research hypothesizes that it is possible to detect an optimal combination of clinical and analytical parameters associated with the development of hypotension or heart failure. By measuring some combination of these parameters in a patient at the start of a dialysis session, the possible occurrence of hypotension during the session could be predicted, helping to reduce its incidence and the need for treatment by medical staff. Identifying the factors that most influence the occurrence of HDI and predicting its occurrence would provide ideal decision support for medical staff. Indeed, determining whether a patient has HDI during HD treatment is a complex task for traditional (statistical) models. However, machine learning (ML)-based models can discover and learn patterns in the data, and are therefore particularly well-suited to address this problem.

This research aims to extract and construct a subset of data consisting of several significantly relevant variables from an extensive database of hemodialysis sessions and blood tests. This subset will be capable of being processed by mass data processing tools to predict the occurrence of hypotension from patterns obtained from an optimal and reduced set of clinical and analytical parameters. Metaheuristic-based techniques will be applied to select an optimal combination of variables from the clinical and analytical blood records of the patient receiving HD treatment. The main contributions of this paper are as follows:

- Use a metaheuristic approach to solve the feature selection problem in the context of hemodialysis and apply two objective functions for this problem.
- Use four metaheuristic algorithms: Particle Swarm Optimization, Grey Wolf Optimizer, Pendulum Search Algorithm, and Whale Optimization Algorithm. In addition, use three classification algorithms, K-Nearest Neighbors, Random Forest, and XGBoost, to evaluate the efficiency of the metaheuristic algorithms.
- Perform in-depth analysis using various performance metrics such as recall, F-score, precision, and number of selected features, graphs, and statistical tests.

As a result of the study and analysis, the XGBoost model demonstrated better performance in the analyzed metrics compared to the other ML models evaluated. It achieved values of around 77% for the recall in the minority class with around 45% of features eliminated in hypotensive patients, minimizing false positives. Consequently, these results suggest that the combination of the use of the metaheuristic PSO, WOA, PSA, GWO, and the ML model XGBoost can predict with high reliability and robustness the occurrence of IDH in patients and, in addition, offers medical staff facilities to support decision-making based on the information inferred by the model itself.

The remainder of this document is structured as follows: Section 2 provides the background and a review of related work. Section 3 introduces the dataset used in this research. In Section 4, we present the metaheuristics and their binarization process for the feature selection task. Section 5 describes the proposed methodology for predicting intradialytic hypotension. Section 6 details the experimental results and evaluates the performance of the different machine learning models. Finally, Section 7 discusses the conclusions drawn from the research and suggests directions for future work.

## 2. Background and Related Works

### 2.1. Soft Computing Techniques in Healthcare

**Figure 1:**
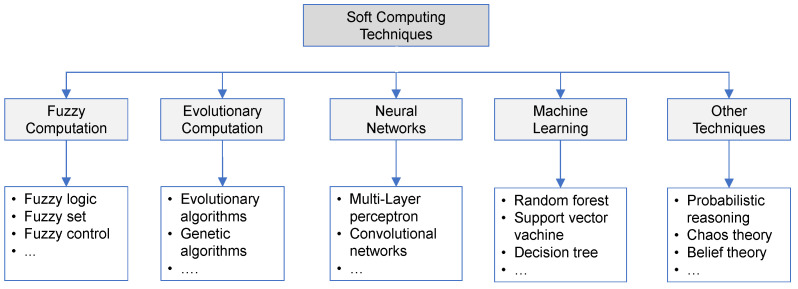
Taxonomy of Soft Computing techniques.

Smart Health, or “Intelligent Health”, greatly interests the scientific and industrial community. Smart Health arises from two already known fronts, the Smart City and e-Health (also known as e-Health), as they aim to improve the health standards of citizens using technological paradigms [7]. Smart Health uses advanced technologies and digital solutions to improve medical care and health in general. The Smart Health dimension generates a considerable amount of information [8] coming from the constant monitoring of patients, who are essential to manage, process and analyze through the implementation of rigorous methods that will allow healthcare personnel to make a more informed decision regarding the care and/or treatment of the patient’s health [9]. For example, digitized clinical history allows medical personnel to keep records of patients’ diseases or pathologies to know the patient’s condition and/or evolution, thus providing effective clinical treatment and care for the patient. Likewise, the application of novel methods allows terminally ill patients, patients with genetic disorders, and/or renal patients to be treated and cared for at home, as the caregiver would make more informed decisions according to the analysis of the patients’ parameters while receiving home care. For example, this is applied to treating patients with in-home palliative care, home MRI, and home hemodialysis, among others [10,11]. Consequently, patients would experience healthier aging or more bearable hemodialysis since family members are present to support them. Considering the above, the use, processing, and analysis of data will contribute significantly to the creation of advanced services of great utility for the various actors involved in the dimensions of CS mentioned above [12]. In this sense, it is necessary to create advanced services through Soft Computing techniques that support intelligent decision-making. These offer a wide range of services with high added value for a better quality of life and welfare of society, as well as improved efficiency and environmental sustainability. On the other hand, the term Soft Computing was proposed by Lofti A. Zadeh, and he points out that Soft Computing is a set of methods or techniques based on imprecision, approximation, and uncertainty to solve complex real-world problems [13,14,15]. Therefore, these techniques can be learned from experience and applied to any problem domain. Because Soft Computing techniques make use of approximate calculations, they offer robust solutions to complex problems. Also, they have a lower computational cost than exact methods [8,15,16]. A characteristic of Soft Computing techniques is that they can be used in combination, offering the possibility of obtaining more reliable results thanks to the characteristics of each of the methods. Soft Computing techniques encompass various advanced algorithms, including fuzzy computing, evolutionary computing, neural networks, machine learning, and deep learning. Figure 1 shows a taxonomy of some of these techniques.

Each technique shown in Figure 1 is used to solve various real-world problems. For example, methods based on fuzzy computing (FC) are employed to solve vague or imprecise problems, i.e., it allows for the transformation of natural language adjectives to quantify the degree of membership of a given dataset [13,17]. Moreover, FC resembles the human ability to make decisions. This is observed in works [18,19] where they make use of fuzzy logic for patient monitoring. Similarly, Soft Computing employs evolutionary computation (EC), inspired by natural evolution. EC is applied to solving optimization problems, and a probabilistic approach is used to solve these problems [17]. Similarly, as in FC techniques, several studies have developed research in the dimensions of Smart Health by applying EC techniques [20,21,22,23,24,25]. On the other hand, machine learning (ML) techniques are widely used due to their potential and versatility in various computer science sectors. These algorithms learn directly from the information they provide, enabling them to extract valuable insights and knowledge from the data itself [26,27,28]. ML algorithms are generally used for classification and/or prediction tasks. As an example, in the study by [29], they used Random Forest and IoT in a healthcare monitoring system. Another Soft Computing technique that recently received much attention is models developed using artificial neural networks (ANNs). The ANN architecture typically consists of an input layer, one or more hidden layers, and an output layer. Examples of ANN architectures are MLP or multilayer perceptron, RBF or radial basis function, SOM networks, or self-organizing maps [17,30,31,32]. Within this framework, the robustness and reliability that ANNs provide today have led to an evolution in ANN architectures, known as deep learning (DL). DL architectures employ more complex processes that enable the capture of intrinsic and abstract relationships in data with greater depth. Some tasks that these DL architectures or models can perform are speech or text recognition, object detection, natural language processing, energy consumption forecasting, and weather forecasting, among others [33]. As a complement, in the Smart Health dimension, researchers have developed DL-based intelligent systems to predict epileptic seizures by analyzing electrocardiogram signals, as well as to predict heart abnormalities [34,35,36,37,38].

### 2.2. Optimization Techniques for IDH Prediction

In healthcare, innovative and efficient solutions for complex problems are crucial [39,40]. Metaheuristics, including genetic algorithms, Particle Swarm Optimization, and ant colony optimization, have found significant applications in this sector. These techniques optimize medical treatments, improve hospital scheduling, and facilitate the analysis of large medical datasets [41,42]. The ability of metaheuristics to find near-optimal solutions in a reasonable amount of time makes them ideal for addressing challenges in healthcare, where quick and accurate decisions can significantly impact patient outcomes.

A specific application of advanced computational techniques in healthcare is the prediction of complications during hemodialysis, such as intradialytic hypotension (IDH). Recent research has developed predictive models that utilize machine learning algorithms and optimization techniques to forecast IDH episodes accurately. These models enable healthcare professionals to anticipate and prevent IDH episodes, enhancing patient safety and well-being during hemodialysis treatments. By combining the power of machine learning with optimization techniques, significant progress has been made in managing and preventing health complications. Various studies have proposed different methodologies to predict and manage IDH episodes, aiming to improve patient outcomes and enhance clinical decision-making.

In [43], the authors present a hybrid model named BSCWJAYA_KELM that integrates serum biomarkers of nutrition with a novel optimization algorithm and machine learning model. By focusing on indicators such as serum uric acid, dialysis vintage, age, diastolic pressure, and albumin, this study aims to improve the accuracy of IDH predictions, ultimately enhancing patient management. In [44], the authors describe developing and validating two AI-based risk models to predict symptomatic IDH during hemodialysis sessions. Their work highlights the importance of personalized approaches in preventing IDH by identifying high-risk patients before the start of dialysis, thereby enhancing treatment efficacy and patient safety. In [45], the authors address the issue of IDH by proposing a predictive model that utilizes features from the photoplethysmography (PPG) signal. Their study emphasizes the potential of PPG in forecasting pre-IDH and IDH episodes, thereby aiding in the prevention of acute hypotension during hemodialysis sessions. In [46], the authors introduce a novel optimization algorithm, the Covariance Matrix-Driven Whale Optimizer with Orthogonal Structure-Assisted Extreme Learning Machine (bCOWOA-KELM), to enhance the prediction accuracy and reliability of IDH. This approach addresses the limitations of existing methods, providing a more solid tool for managing hemodialysis complications.

In another study [46], the authors explore ensemble machine learning techniques to predict various hemodialysis complications, including IDH. The objective is to develop high-performance models for early prediction, thus improving patient care and reducing the incidence of early dialysis session terminations. In [47], the authors discuss the development of an early alert system for predicting IDH before the initiation of hemodialysis. By employing artificial intelligence (AI) and machine learning techniques, their study aims to identify high-risk patients early, allowing for timely interventions and better management of IDH. In [48], the authors propose an optimized machine learning framework for predicting IDH, with a focus on indices related to chronic kidney disease–mineral and bone disorders (CKD-MBDs). Their model aims to predict IDH episodes effectively, thereby supporting clinical decision-making and reducing the risk of severe complications during hemodialysis.

These studies collectively contribute to IDH prediction and management knowledge, utilizing advanced machine learning algorithms and AI techniques to improve patient outcomes during hemodialysis.

In summary, metaheuristics, machine learning, and optimization techniques are pivotal in advancing healthcare solutions [49,50]. Their applications enhance operational efficiencies and have a direct impact on patient care and treatment outcomes.

## 3. Hypotension Dataset

### 3.1. Digitized Clinical Database of Hemodialysis Patients

**Figure 2:**
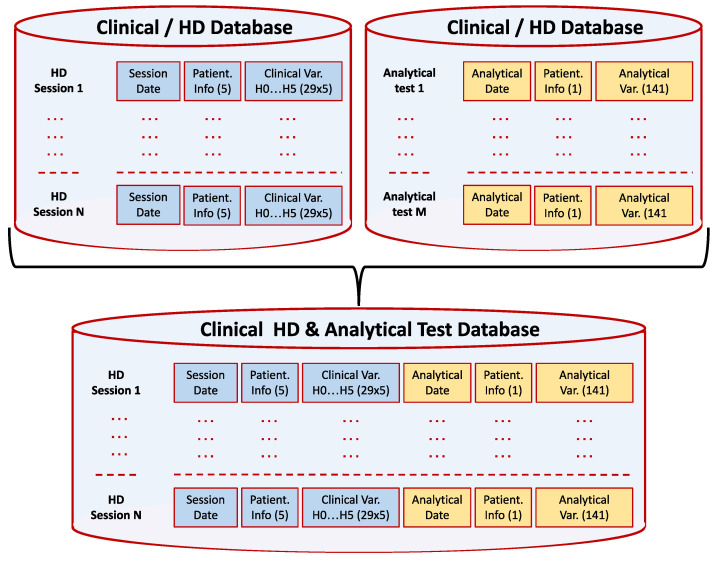
Digitized clinical database of hemodialysis patients.

Our research is based on two extensive databases containing the medical information of 758 patients undergoing hemodialysis sessions. These data were collected at the Hospital *Príncipe de Asturias*, Madrid, Spain, over almost four years, from 1 January 2016 to 30 October 2019. This dataset contains records relating to clinical variables of patients who received HD treatment between January 2016 and October 2019. The first database collected each patient’s clinical data for 98,015 hemodialysis sessions of about five hours’ duration, which are labeled as “Hour 0”, “Hour 1”, “Hour 2”, “Hour 3”, “Hour 4”, and “Hour 5”. This first data source contains the following patient and session identification data: patient identifier, gender, and age; whether the patient is hypertensive or diabetic; and the date of the session. The remaining 29 variables contain the sequence of the different clinical parameters measured at the corresponding time of the session. The number of variables associated with a dialysis session is 180. Additionally, a second database containing data from the same patients during the same period was available, with the variables and corresponding values of the information extracted from the hospital laboratory’s blood analyses. This second database contains the values of 141 variables. The total set of variables related to patient, dialysis session, and blood test data amounts to 221. The hospital’s ethics committee approved the collection of all these records, and the database was completely anonymized. From the two databases above, a new database was constructed incorporating the values of the blood test variables and linking them to the nearest hemodialysis session, either on the same date or immediately before. Figure 2 shows the unification process of both datasets. It should be noted that the different analytical variables were not obtained in the same tests and with the same periodicity.

### 3.2. Determination of IDH in the HD Session

**Figure 3:**
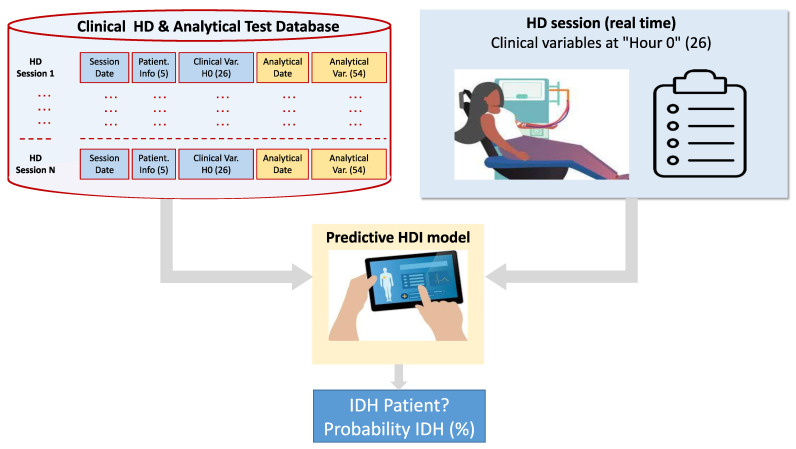
Predictive model at the start of the hemodialysis session.

**Figure 4:**
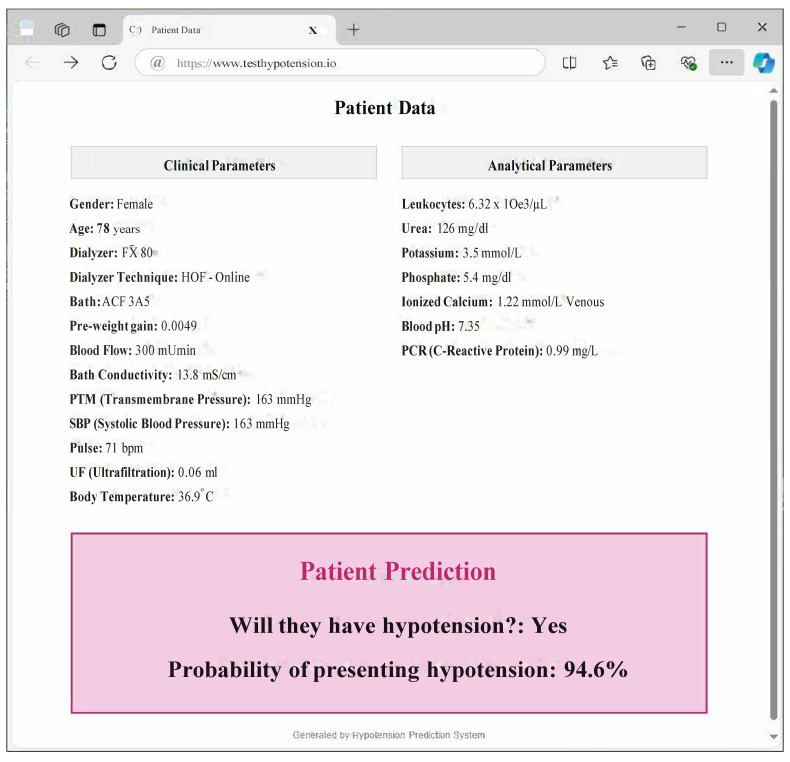
HDI prediction system mockup.

The potential hypotension that may occur during a hemodialysis session is determined by medical judgment, calculated from the systolic blood pressure measurements taken at each of the five hours of the session. If any of the pressures measured at “Hour 2”, “Hour 3”, and “Hour 4” is lower by at least 20 mL than any of the pressures measured at “Hour 0” and “Hour 1”, then hypotension is present, quantified by the Hypotension Measurement (HYM) as the difference in systolic blood pressures; otherwise, there is no hypotension. Once the possible hypotension values for all sessions have been calculated, the HYM variable and the binary variable for the presence of IDH are incorporated into the database. Subsequently, a new database is generated which only takes into account the values of the clinical variables at the beginning of the HD session, the so-called “Hour 0”, given that the final objective of this work is to develop a predictive system capable of accurately anticipating the possible future appearance of hypotension during the dialysis session, as indicated by [51,52]. Figure 3 and Figure 4 show the hypotension warning process prior to Hour 0 and the mockup of the application visible to medical staff respectively.

### 3.3. Data Processing and Variables Considered in the Clinical Study

After obtaining the integrated database, the medical staff, utilizing their experience and clinical knowledge, identified a set of 80 variables (26 related to the patient and the dialysis session, and 54 to the analytical tests) to be considered for developing the predictive model. Subsequently, as is common in data science projects, pre-processing the data is essential before proceeding to the modeling phase. In this case, data cleaning tasks were performed after integrating and merging data from different sources to identify and correct potential errors. An analysis of the variables was conducted to identify potential quality issues, and cleaning tasks were performed to ensure a consistent dataset by correcting incorrect, atypical, or missing data ([53,54,55]). This analysis revealed that many dialysis sessions or analytical tests lacked quality data. As a result, the medical team retained the most significant variables and excluded patients and dialysis sessions with insufficient quality data. The final dataset comprised 68,574 samples, divided into two classes: the majority class (non-hypotensive during dialysis sessions) with 48,764 cases (71.11% of the dataset) and the minority class (hypotensive during dialysis sessions) with 19,810 cases (28.89%). These samples comprise 328 patients and 68,574 dialysis sessions, ensuring that both the cohort size and the number of sessions are representative of the population under study. After pre-processing, the dataset was expanded to 87 features, derived from the original 71 features through the one-hot encoding of 3 variables. Table 1 provides a summary regarding the IDH dataset, **Instances** refers to the total number of instances in the dataset. **Labels** categorizes the dataset into two classes **NH**, representing dialysis sessions where no hypotension occurred, and **H**, representing sessions where hypotension was observed. **Features** includes two stages of feature processing: **Pre.** for the number of features before pre-processing, and **Post** for the number of features after pre-processing.

Table 2 presents the classification results of three machine learning classifiers (XGBoost, Random Forest (RF), and K-Nearest Neighbors (KNN)) applied to the dataset without any optimization or feature selection processes. These results reflect the raw performance of the predictive models, with no techniques employed to reduce the number of features or address the class imbalance in the data. The table includes several key performance metrics: macro-averaged F-score (fs_macro), recall (r_macro), and precision (p_macro) for all classes, as well as these metrics for the minority class (fs_min, r_min, p_min) and the majority class (fs_may, r_may, p_may).

The macro-averaged metrics provide an overall evaluation across both classes. In contrast, the minority and majority class-specific metrics allow for a more detailed analysis of the classifier’s performance in handling the imbalanced dataset. As seen, XGBoost generally achieves the highest F-scores, recall, and precision, particularly in the majority class. In contrast, the performance of the minority class is notably lower across all classifiers, reflecting the challenge posed by class imbalance.

## 4. Optimization of Relevant Feature Selection for the Predictive Model

This section develops a method demonstrating the great capability and versatility of models based on Soft Computing techniques to adapt to the requirements of highly reliable clinical predictive systems. This allows for providing highly relevant and valuable information for medical decision-makers.

The feature selection (FS) problem is a multi-objective combinatorial optimization issue that stems from the need to eliminate irrelevant and redundant information from datasets used in machine learning training, as such information is detrimental to the learning tasks of prediction or classification algorithms.

In its mathematical definition, FS assumes a dataset *O* such as the original dataset, which contains a *o* number of *f* features, such that $O=\{f_{1},f_{2},f_{3},\ldots ,f_{o}\}$. The objective of the problem is to select the best subset of features $B=\{f_{1},f_{2},f_{3},\ldots ,f_{b}\}$ with b<o so that the features belonging to the selected subset are the most representative of the set of original data.

**Figure 5:**
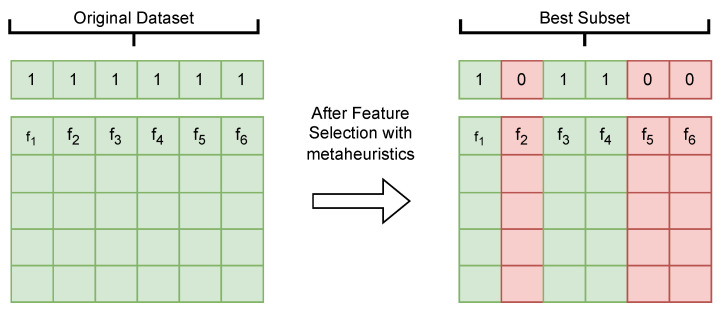
Binary representation of vectors *S* and *D*.

Internally, FS has a binary domain; the solutions are represented by ones and zeros, where a one indicates that the feature is included in the data subset, while a zero indicates the opposite. Figure 5 shows the representation of the solutions for the feature selection problem. In this example, it is shown that the original dataset is composed of six features. Therefore, the solution is [1,1,1,1,1,1]. After applying the metaheuristics, we will obtain the best subset of features composed of the features $f_{1},f_{3},$ and $f_{4}$. Therefore, the solution is [1,0,1,1,0,0].

The objective function to optimize within the feature selection problem is represented in various ways, with the weighted multi-objective function being the most commonly used, as noted by [56]. In this way, the objective function *Z* is calculated as follows:

(1) $$min\;Z=\alpha \cdot metric+\beta \cdot \frac{\left|R\right|}{\left|N\right|}$$

where metric corresponds to a performance metric obtained from the machine learning technique, |R| is the number of selected features, and |N| is the total number of features, with α∈[0,1] and β=(1−α) parameters that regulate the importance of the quality of the results and the size of the subset, respectively.

### 4.1. Two−Step Techniques

**Figure 6:**
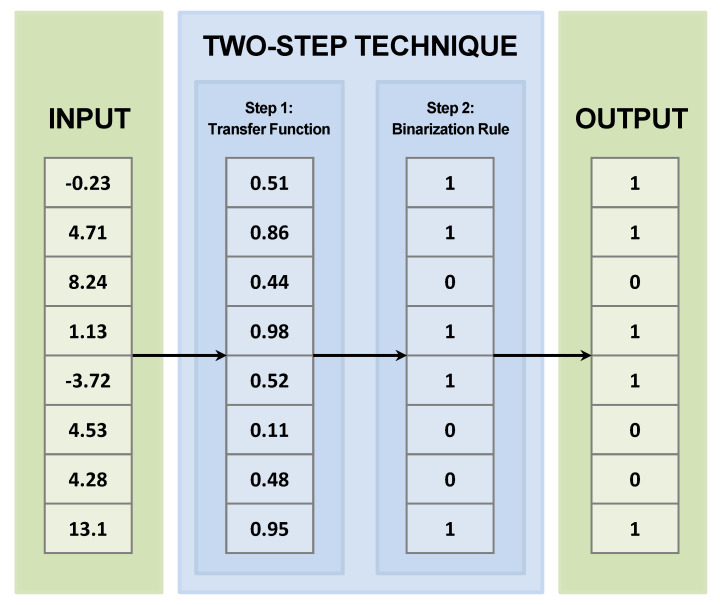
Two−step technique.

A review of the literature reveals a great diversity of implemented metaheuristics. The work carried out in [57] summarizes around 500 metaheuristics, highlighting that most of them are continuous population metaheuristics. On the other hand, there is significant interest in using continuous metaheuristics to solve binary combinatorial problems, particularly in relation to the feature selection problem, as highlighted by [56,58]. Figure 6 provides an overview of the two-step technique.

#### 4.1.1. Transfer Function

A transfer function aims to transfer the values assigned to the decision variables from the R domain to the [0,1] domain. The first transfer function proposed in the literature within the context of metaheuristics was in 1997 with the presentation of the first binary metaheuristic, Binary Particle Swarm Optimization (BPSO), as noted by [59].

As research into binarization methods has deepened, [58] proposed new transfer functions in the literature, including the S-shaped and V-shaped transfer functions introduced by [60].

**Figure 7:**
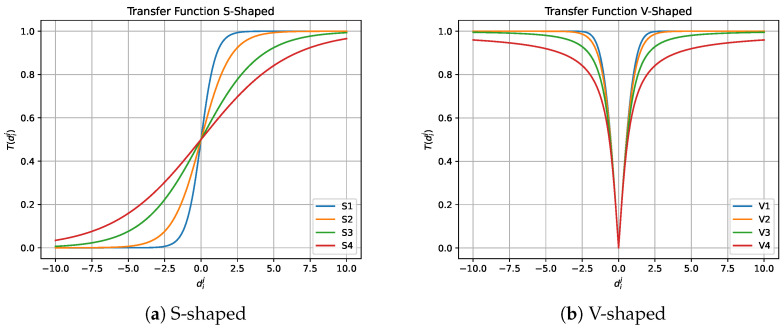
S-shaped and V-shaped transfer functions.

Table 3 and Figure 7 show the S-shaped transfer functions and V-shaped transfer functions found in the literature. The notation $d_{i}^{j}$ observed in Table 3 corresponds to the continuous value of the j−th dimension of the i−th individual resulting after the perturbation performed by the continuous metaheuristic.

#### 4.1.2. Binarization Rule

The second step is discretizing the value transferred from step 1 by applying a binarization rule. Various rules described in the scientific literature by [61,62] can be utilized for this binarization process. Table 4 shows the five binarization rules found in [61].

The notations $X_{i}^{j}$ and $X_{Best}^{j}$ observed in Table 4 correspond to the j-th dimension binary value of the i-th current individual and the j-th dimension binary value of the best solution, respectively.

Algorithm 1 shows the general process of the two-step technique, where the process is performed for each solution for each dimension.
**Algorithm 1** Two-step technique.**Input:** Continuous population $X=\{X_{1},X_{2},\ldots ,X_{pop}\}$**Output:** Binary population $X^{\prime }=$$\left\{X_{1}^{\prime },X_{2}^{\prime },\ldots ,X_{pop}^{\prime }\right\}$1:**for** i=1 **to** pop **do**                        ▹ Binarization Process2:     **for** j=1 **to** dim **do**3:           Get $T\;(d_{i}^{j})$ by applying transfer function4:           Get $X_{new}^{j}$ by applying Binarization Rule5:      **end for**6:**end for**

### 4.2. Binary Particle Swarm Optimization

Particle Swarm Optimization is considered one of the first population metaheuristics presented in the literature by [63]. This metaheuristic simulates the collective behavior of swarms such as birds or fish. This algorithm is one of the most studied and referenced in the field of metaheuristics, demonstrating its versatility in different optimization problems, as highlighted by [57,58]. The following equation gives the equation of motion:

(2) $$X_{i}\left(t+1\right)=X_{i}\left(t\right)+v_{i}\left(t+1\right)$$

where $X_{i}\left(t\right)$ is the current position of particle *i*, and $X_{i}\left(t+1\right)$ is the new position of particle *i* after updating the velocity $v_{i}\left(t+1\right)$. The velocity $v_{i}\left(t+1\right)$ is calculated as follows:

(3) $$v_{i}\left(t+1\right)=w\cdot v_{i}\left(t\right)+c_{1}\cdot r_{1}\cdot \left(p_{Best}-X_{i}\left(t\right)\right)+c_{2}\cdot r_{2}\left(g_{Best}-X_{i}\left(t\right)\right)$$

where $c_{1}$ and $c_{2}$ are the acceleration coefficients that determine the influence of individual and collective knowledge on the motion of the particle, $r_{1}$, and $r_{2}$, are random numbers between [0,1], $p_{Best}$ is the personal best position of particle *i*, $g_{Best}$ is the global best position found by the entire swarm up to that moment, $X_{i}\left(t\right)$ is the current position of particle *i*, $v_{i}\left(t\right)$ is the current velocity of particle *i*, and *w* is the inertia factor that controls the influence of the previous velocity on the new velocity.

The inertia factor (*w*) plays an important role in the metaheuristic since it allows for the balancing of exploration and exploitation. This inertia factor is calculated as follows:

(4) $$w=w_{max}-\frac{w_{max}w_{min}}{maxIter}\cdot t$$

where $w_{max}$ corresponds to the maximum inertia factor, $w_{min}$ corresponds to the minimum inertia factor, maxIter corresponds to the maximum number of iterations, and *t* corresponds to the current iteration. This equation allows for a linear decrease from the maximum inertia factor ($w_{max}$) to the minimum inertia factor ($w_{min}$).

This metaheuristic was designed to solve continuous optimization problems. Thus, the creators of PSO,  [59], proposed the binary version of PSO, and Algorithm 2 shows its behavior.
**Algorithm 2** Binary Particle Swarm Optimization. **Input:** The population X=$\left\{X_{1},X_{2},\ldots ,X_{i}\right\}$ **Output:** The updated population $X^{\prime }=$$\left\{X_{1}^{\prime },X_{2}^{\prime },\ldots ,X_{i}^{\prime }\right\}$ and $X_{\alpha }$ 1:**Initialize binary random population X** 2:Evaluate the objective function of each individual and save the $p_{best}$ of each solution 3:Identify the $g_{Best}$ in the population 4:Define $c_{1}$, $c_{2}$, $w_{max}$, and $w_{min}$ 5:**for** iteration (t) **do** 6:      Update *w* using Equation (4) 7:      **for** solution(i) **do** 8:            Update $v_{i}$ using Equation (3) 9:            Update $X_{i}$ using Equation (2)10:      **end for**11:      **Binarization of population X using Algorithm 1**12:      Evaluate the objective function of each individual and save the $p_{best}$ of each solution13:      Update $g_{Best}$14:**end for**15:Return ($g_{Best}$)

### 4.3. Binary Grey Wolf Optimizer

The Grey Wolf Optimizer was proposed by [64] and simulates the social hierarchy and hunting behavior of grey wolves in the wild. The wolf hierarchy is composed of the alpha wolf (α), who is the pack leader and is represented by the best solution in the population; the beta wolf (β), who can assume leadership in the absence of alpha and is represented by the second best solution in the population; the delta wolf (δ), who helps the alpha and beta wolves control the pack and is represented by the third best solution in the population; and the omega wolves (ω), who are the lowest-ranking wolves and are represented by the rest of the population. Stalking and surrounding behavior is modeled as follows:

(5) $$\vec{X}\left(t+1\right)=\frac{{\vec{X}}_{1}+{\vec{X}}_{2}+{\vec{X}}_{3}}{3}$$

where ${\vec{X}}_{1}$, ${\vec{X}}_{2}$, and ${\vec{X}}_{3}$ are the updated positions of the wolves based on the positions of the alpha wolf (${\vec{X}}_{\alpha }$), beta wolf (${\vec{X}}_{\beta }$), and delta wolf (${\vec{X}}_{\delta }$)

(6) $$\begin{array}{c} {\vec{X}}_{1}={\vec{X}}_{\alpha }-\left({\vec{A}}_{1}\cdot {\vec{D}}_{\alpha }\right)\\ {\vec{X}}_{2}={\vec{X}}_{\beta }-\left({\vec{A}}_{2}\cdot {\vec{D}}_{\beta }\right)\\ {\vec{X}}_{3}={\vec{X}}_{\delta }-\left({\vec{A}}_{3}\cdot {\vec{D}}_{\delta }\right) \end{array}$$

where ${\vec{D}}_{\alpha }$, ${\vec{D}}_{\beta }$, and ${\vec{D}}_{\delta }$ corresponds to the distance between the position of the wolf *X* that we are updating and the wolves ${\vec{X}}_{\alpha }$, ${\vec{X}}_{\beta }$, and ${\vec{X}}_{\delta }$.

(7) $$\begin{array}{c} {\vec{D}}_{\alpha }=\left|{\vec{C}}_{1}\cdot {\vec{X}}_{\alpha }-\vec{X}\right|\\ {\vec{D}}_{\beta }=\left|{\vec{C}}_{2}\cdot {\vec{X}}_{\beta }-\vec{X}\right|\\ {\vec{D}}_{\delta }=\left|{\vec{C}}_{3}\cdot {\vec{X}}_{\delta }-\vec{X}\right| \end{array}$$

*A* and *C* are coefficients that play different roles in metaheuristic behavior. On the one hand, *C* interferes with the direction and intensity of the position adjustment relative to the best solutions, while *A* plays a vital role in the exploration–exploitation balance.

When |A|>1, the wolf moves away from the prey, allowing the exploration of new areas of the search space. On the other hand, when |A|<1, the wolf approaches the prey, promoting exploitation. The coefficient *a* decreases linearly from 2 to 0 during the optimization, meaning that the value of *A* also decreases, gradually shifting the focus of the algorithm from exploration to exploitation.

(8) $$\begin{array}{c} \vec{A}=2\vec{a}\cdot {\vec{r}}_{1}-\vec{a}\\ \vec{C}=2\cdot {\vec{r}}_{2}\\ \vec{a}=2-\frac{2\cdot t}{maxIter} \end{array}$$

This metaheuristic was also designed to solve continuous problems. Therefore, we must incorporate the two-step technique to solve binary combinatorial problems. Such an adaptation was proposed in [65], and Algorithm 3 shows its behavior.
**Algorithm 3** Binary Grey Wolf Optimizer. **Input:** The population X=$\left\{X_{1},X_{2},\ldots ,X_{i}\right\}$ **Output:** The updated population $X^{\prime }=$$\left\{X_{1}^{\prime },X_{2}^{\prime },\ldots ,X_{i}^{\prime }\right\}$ and $X_{\alpha }$ 1:**Initialize binary random population X** 2:Evaluate the objective function of each individual 3:Identify the $X_{\alpha }$, $X_{\beta }$, and $X_{\delta }$ in the population 4:**for** iteration (t) **do** 5:      **for** solution(i) **do** 6:            Update *a*, *A*, and *C* using Equation (8) 7:            Update $X_{1}$, $X_{2}$, and $X_{3}$ using Equation (6) 8:            Update the position of $X_{i}^{t}$ using Equation (5) 9:      **end for**10:    **Binarization of population X using Algorithm 1**11:    Evaluate the objective function of each individual12:    Update $X_{\alpha }$, $X_{\beta }$, and $X_{\delta }$13:**end for**14:Return ($X_{\alpha }$)

### 4.4. Binary Pendulum Search Algorithm

The Pendulum Search Algorithm is a new population-based metaheuristic created in 2022 by Nor Azlina Ab. It was inspired by [66] and the harmonic motion of the simple pendulum. The search agents are initialized randomly, and their position is updated using Equation (9).

(9) $$X_{i,j}^{t}=X_{i,j}^{t}+pend_{i,j}^{t}\cdot \left(Best_{j}-X_{i,j}^{t}\right)$$

where $X_{i,j}^{t}$ is the position of the *i*-th solution in the *j*-th dimension in *t*-th iteration, $Best_{j}$ it is the position of the best solution in the *j*-th dimension in the *t*-th iteration, and $pend_{i,j}^{t}$ is a parameter which is calculated as follows:

(10) $$pend_{i,j}^{t}=2\cdot e^{\left(-t/tmax\right)}\cdot cos\left(2\cdot \pi \cdot rand\right)$$

where *t* is the current iteration, tmax is the maximum number of iterations and rand is a uniform random number between [0,1].

This metaheuristic was also designed to solve continuous problems. Therefore, we must incorporate the two-step technique to solve binary combinatorial problems. Such an adaptation was proposed in [67], and Algorithm 4 shows its behavior.
**Algorithm 4** Binary Pendulum Search Algorithm. **Input:** The population X=$\left\{X_{1},X_{2},\ldots ,X_{i}\right\}$ **Output:** The updated population $X^{\prime }=$$\left\{X_{1}^{\prime },X_{2}^{\prime },\ldots ,X_{i}^{\prime }\right\}$ and Best 1:**Initialize binary random population X** 2:Evaluate the objective function of each individual 3:Identify the best individual in the population (Best) 4:**for** iteration (t) **do** 5:      **for** solution(i) **do** 6:            **for** dimension(j) **do** 7:                  Update $pend_{i.j}^{t}$ by Equation (10) 8:                  Update the position of $X_{i,j}^{t}$ using Equation (9) 9:            **end for**10:      **end for**11:      **Binarization of population X using Algorithm 1**12:      Evaluate the objective function of each individual13:      Update Best if there is a better solution14:**end for**15:**return** the updated population $X^{\prime }$ where Best is the best result

### 4.5. Binary Whale Optimization Algorithm

The Whale Optimization Algorithm (WOA) is inspired by the hunting behavior of humpback whales, in particular how they search (Section 4.5.1), encircle (Section 4.5.2) and attack (Section 4.5.3) their prey with a technique known as “bubble nets”. This algorithm was devised by [68].

#### 4.5.1. Searching for Prey

Searching for prey is related to the exploration phase of the search space. To ensure a good exploration, the value of A is used. When |A|>1, whales move away from a known solution, allowing a global search for space exploration. This behavior is modeled as follows:

(11) $$\begin{array}{cc} {\vec{X}}_{i}^{t+1} & =\vec{X_{rand}^{t}}-\vec{A}\cdot \vec{D}\\ \vec{D} & =|\vec{C}\cdot \vec{X_{rand}^{t}}-{\vec{X}}_{i}^{t}| \end{array}$$

where $\vec{A}$ and $\vec{C}$ are coefficient vectors, *t* denotes the current iteration, and $\vec{X_{rand}^{t}}$ is a random whale in the population. The coefficient vectors $\vec{A}$ and $\vec{C}$ can be computed according to Equations (12) and (13):

(12) $$\vec{A}=2\vec{a}\cdot \vec{r}-\vec{a}$$

(13) $$\vec{C}=2\cdot \vec{r}$$

where $\vec{a}$ decreases linearly from 2 to 0 over the course of the iterations (both in the exploration and exploitation phases) and $\vec{r}$ corresponds to a random vector of values between [0,1].

#### 4.5.2. Encircling the Prey

The first exploitation operator is related to the encircling prey. This occurs when a whale approaches the best solution found in the population so far. It is modeled using the following equations:

(14) $$\begin{array}{cc} {\vec{X}}_{i}^{t+1} & =\vec{X^{*}}-\vec{A}\cdot \vec{D}\\ \vec{D} & =|\vec{C}\cdot \vec{X^{*}}-{\vec{X}}_{i}^{t}| \end{array}$$

where $\vec{X^{*}}$ is the best whale obtained so far and ${\vec{X}}_{i}^{t}$ is the i-th whale in the t-th iteration. The vectors $\vec{A}$ and $\vec{C}$ are calculated as shown in Equations (12) and (13).

#### 4.5.3. Spiral Movement

The second exploitation operator is related to spiral motion. This occurs when whales move towards the best known solution in a spiral-shaped path. The following equation models this:

(15) $$\begin{array}{cc} {\vec{X}}_{i}^{t+1} & =\vec{D^{\prime }}\cdot e^{bl}\cdot \cos\left(2\pi l\right)+\vec{X^{*}}\\ \vec{D^{\prime }} & =|\vec{X^{*}}-{\vec{X}}_{i}^{t}| \end{array}$$

where $\vec{D^{\prime }}$ is the distance of the i-th whale from the prey (the best solution obtained so far), and *b* is a constant to define the shape of the logarithmic spiral. *l* is a random number between [−1,1].

In nature, humpback whales perform the process of encircling the prey and spiral movement simultaneously. To model this simultaneous behavior, there is a 50% chance of choosing between the prey encircling mechanism and the spiral model. The mathematical model is as follows:

(16) $$\begin{array}{cc} \; & {\vec{X}}_{i}^{t+1}=\left\{\begin{array}{cc} \vec{X^{*}}-\vec{A}\cdot \vec{D} & \mathrm{If}\;p<0.5\\ \vec{D^{\prime }}\cdot e^{bl}\cdot \cos\left(2\pi l\right)+\vec{X^{*}} & \mathrm{If}\;p\geq 0.5 \end{array}\right. \end{array}$$

This metaheuristic was also designed to solve continuous problems. Therefore, we must incorporate the two-step technique to solve binary combinatorial problems. This adaptation was proposed in [65], and Algorithm 5 shows its behavior.
**Algorithm 5** Binary Whale Optimization Algorithm. **Input:** The population X=$\left\{X_{1},X_{2},\ldots ,X_{i}\right\}$ **Output:** The updated population $X^{\prime }=$$\left\{X_{1}^{\prime },X_{2}^{\prime },\ldots ,X_{i}^{\prime }\right\}$ and $X^{*}$ 1:**Initialize binary random population X** 2:Evaluate the objective function of each individual 3:Identify the best individual in the population ($X^{*}$) 4:**for** iteration (t) **do** 5:      **for** solution(i) **do** 6:            Update a,A,C,l and *p* 7:            **if** p<0.5 **then** 8:                 **if** |A|<1 **then** 9:                       Apply Equation (14)10:               **else**11:                     Apply Equation (11)12:               **end if**13:            **else**14:               Apply Equation (15)15:            **end if**16:      **end for**17:      **Binarization of population X using Algorithm 1**18:      Evaluate the objective function of each individual19:      Update $X^{*}$ if there is a better solution20:**end for**21:Return ($X^{*}$)

## 5. Enhanced Prediction of IDH Through ML and Biomarker Analysis

### 5.1. Addressing the Imbalance in the Dataset

As mentioned in Section 3 and visible in the preliminary results in Table 2, data imbalance significantly harms the performance of the machine learning model for the minority class.

To address the dataset imbalance, several machine learning techniques were tested. These techniques were categorized into three main approaches: undersampling, oversampling, and hybrid. Undersampling techniques aim to remove samples from the majority class to bring them closer to the number of minority samples. Oversampling techniques aim to create synthetic samples from the minority class to balance it with the majority class. Finally, hybrid techniques mix both oversampling and undersampling techniques. Currently, Python 3.13.2 is a very popular programming language in machine learning due to its access to multiple libraries related to this field. Data imbalance techniques are no exception, and the Imbalanced-learn library, developed by [69], is the tool.

We have performed preliminary experiments with different algorithms for each type of swing. For undersampling, two methods were applied: (i) Random Undersampler, which reduces the majority class by randomly selecting a subset of its instances, and (ii) NearMiss, which selects instances from the majority class that are closest to the minority class based on distance measures. For oversampling, two techniques were used: (i) Random Oversampler, which increases the size of the minority class by randomly duplicating instances, and (ii) SMOTE (Synthetic Minority Oversampling Technique), which generates synthetic instances for the minority class. Additionally, a hybrid technique, SMOTEENN, was tested, which combines SMOTE for oversampling and Edited Nearest Neighbors (ENN) for undersampling. The results indicate that the best data balancing technique for our dataset is the Random Undersampler.

### 5.2. Construction of Objective Function

Our proposal incorporates metaheuristics for feature selection to improve the performance of classification models that seek to predict intradialytic hypotension during dialysis treatment.

As mentioned in Section 4, metaheuristics require an objective function to perform the optimization process. Although Equation (1) defines the objective function, obtaining the metrics incorporates several steps. First, we need to work with the data to maintain data balance, perform cross-validation to avoid data bias, and apply a classifier to obtain the performance metrics. Given this, Algorithm 6 shows how we calculate the objective function in our proposal.
**Algorithm 6** Objective function. **Input:** Selected features and dataset **Output:** Objective function and performance metrics 1:Filter the original dataset with the selected features 2:Apply KFold technique for cross-validation 3:**for** each fold **do** 4:      Apply balancing technique for training fold 5:      Normalize training fold and testing fold using MinMax technique 6:      Train the model with the classification algorithm 7:      Predict with the trained model from the testing fold 8:      Compare predicted observations with test fold 9:      Save performance metrics from the fold10:**end for**11:Calculate the average of the metrics across the KFold12:Apply Equation (1)13:Return average metrics and evaluate the objective function

### 5.3. Selection of Classifiers

In our proposal, we have considered three different classification algorithms for line 6 of Algorithm 6. The classifiers used were K-Nearest Neighbors (KNN), Random Forest (RF), and XGBoost (XG). KNN was chosen because of its wide use in the literature, as mentioned by [70], and because it is a distance-based classifier, while RF and XGBoost were chosen for their frequent use as decision tree- and boosting-type classifiers, respectively, as described by [56].

### 5.4. Metaheuristics for Feature Selection

The metaheuristics used in this study include Particle Swarm Optimization (PSO), Grey Wolf Optimizer (GWO), Pendulum Search Algorithm (PSA), and Whale Optimization Algorithm (WOA) as described in Section 4. As discussed in the articles by [57,66], the algorithms were selected for the following reasons: PSO for its strong performance in various optimization tasks, GWO for its growing popularity in recent years, WOA for its relevance in the literature, and PSA for being a parameter-free metaheuristic, simplifying the configuration process. In addition, we have used different metaheuristics, and not just one, based on the No Free Lunch Theorem ([71,72,73]), which tells us that there is no optimization algorithm capable of solving all existing optimization problems.

**Figure 8:**
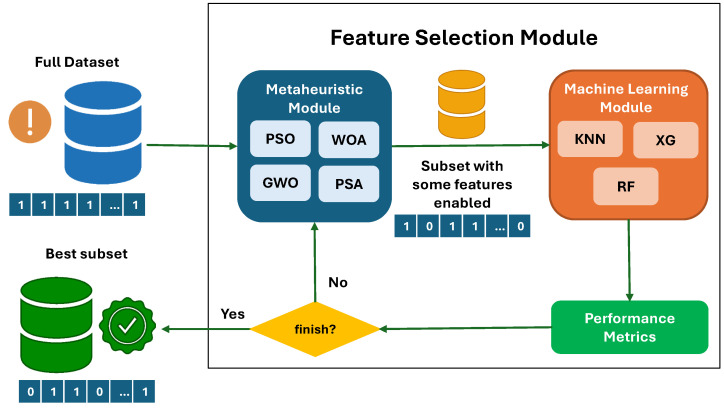
Enhanced prediction of IDH through ML and biomarker analysis.

As mentioned in Section 4, the metaheuristics we have used must be binarized to solve binary combinatorial problems. In this case, we have used the two-step technique presented in Algorithm 1, where we have used the S4 function as the transfer function and the standard rule as the binarization rule. This selection was based on previous work in [67]. Thus, our complete proposal is summarized by Figure 8.

## 6. Results

This section presents the results of the experiments that evaluate the performance of the proposed approach. We describe the experimental setup, including the datasets, evaluation metrics, and metaheuristic configurations. Additionally, we detail the hardware used and the number of experiments performed. Finally, we compare and discuss the results obtained under different configurations.

### 6.1. Experiment Configuration

#### 6.1.1. Sampling Parameters

Table 5 presents the classification results of three different classifiers (KNN, Random Forest, and XGBoost) using the Random Undersampler technique with varying sampling parameters. The classifiers are evaluated based on several performance metrics: macro F-score (f1_m), recall (r_m), and precision (p_m), as well as F-scores, recall, and precision for both the minority class (f1_min, r_min, p_min) and the majority class (f1_may, r_may, p_may). Additionally, the computation time for each configuration is reported.

From the results described in Table 5, it can be noted that across all parameters, the effect of the sampling parameter is that as the parameter decreases from 1.0 to 0.6, all classifiers show a slight increase in the macro F-scores. For instance, the f1_m of XGBoost increases from 0.725 at a parameter value of 1.0 to 0.745 at a parameter value of 0.6, suggesting an improvement in overall performance. However, this improvement comes at the expense of the minority class recall (r_min), which is critical for correctly predicting hypotensive episodes and reducing false positives. For XGBoost, r_min decreases from 0.766 at a parameter of 1.0 to 0.64 at a parameter of 0.6, indicating a significant reduction in the model’s ability to detect minority class instances. Similarly, Random Forest shows a considerable drop in r_min from 0.738 at 1.0 to 0.593 at 0.6. Therefore, although lower parameter values improve macro metrics, they negatively affect the minority class performance, going against the primary goal of accurately predicting hypotensive episodes.

Based on these findings, a classifier with a parameter value of 1.0 is recommended. This classifier provides the highest recall for the minority class, ensuring the best performance in minimizing false positive errors and correctly predicting hypotensive episodes.

#### 6.1.2. Classifiers Parameters

In this proposal, we have used three classifiers: KNN, Random Forest, and XGBoost. For the first two algorithms, the scikit-learn library proposed by [74] was used, while for XGBoost, the XGBoost library proposed by [75] was implemented. Both libraries are coded in Python. As the main objective is to improve the performance of the classifiers, we have used the default parameters of each classifier provided by each library.

#### 6.1.3. Metaheuristic Configuration

The parameters of each metaheuristic used in our experimentation were obtained from the recommendations made by the authors of the previously mentioned studies. The details of the parameters are shown in Table 6.

#### 6.1.4. Objective Function

The objective function used to train the model is a weighted multi-objective function that aims to minimize the classification error and the number of selected features. The weighted multi-objective function can be defined as

(17) $$\mathrm{minimize}\;z=\alpha \cdot f_{1}+\left(1-\alpha \right)\cdot f_{2}$$

where

- α is a weight parameter set to 0.99 in this experiment.
- $f_{1}$ represents the classification error metric.
- $f_{2}$ represents the proportion of selected features.

We define two specific objective functions to evaluate the performance of the model:

- **Objective Function 1 (OF1)**
  (18) $$\mathrm{minimize}\;z_{1}=0.99\cdot \left(1-\mathrm{Recall}\;\mathrm{Macro}\right)+0.01\cdot \frac{\mathrm{NSF}}{\mathrm{TNF}}$$
  Recall macro is the simple recall average for both classes, representing the ratio of false negatives overall, NSF is the number of selected features, and TNF is the total number of features.
- **Objective Function 2 (OF2)**
  (19) $$\mathrm{minimize}\;z_{2}=0.99\cdot \left(1-\mathrm{Recall}\;\mathrm{Minority}\right)+0.01\cdot \frac{\mathrm{NSF}}{\mathrm{TNF}}$$
  where recall minority represents the ratio of false negatives in the minority class, and as in Objective Function 1, NSF is the number of selected features, and TNF is the total number of features.

#### 6.1.5. Experimentation Environment

A total of 24 experiments were conducted, accounting for combinations of metaheuristics (4), classifiers (3), and objective functions (2). Each experiment was repeated 31 times to ensure robust results and account for the metaheuristics’ stochastic nature. The results were averaged, and the best outcomes were compared across these repetitions. The different metrics used are described next.

The experiments were executed on a machine equipped with an Intel Core i7 processor and 16 GB of RAM. They were run on a Windows operating system, ensuring consistent trial performance. Regarding the coding of the experiments, the following Python libraries were used: NumPy==1.24.4, SciPy==1.15.2, Scikit-learn==1.1.1, Pandas==1.4.3, Matplotlib==3.5.2, Seaborn==0.11.2, XGBoost==1.7.3, and Imbalanced-learn==0.11.0.

To ensure a robust evaluation of the classifiers, we employed k-fold cross-validation in all experiments with (k=5).

### 6.2. Evaluation Criteria

For the evaluation, we used five types of metrics. The first one is related to the evaluation in the objective function, the second one is related to macro metrics, the third one is related to majority class metrics, the fourth one is related to minority class metrics, and the fifth one is related to the number of selected features. The mathematical formulations of each one are detailed below.

#### 6.2.1. Macro Metrics

As defined in the scikit-learn libraries proposed by [74], macro metrics calculate metrics for each label and find their unweighted mean. This does not take label imbalance into account.

- **F-score (f1_m)**: The F-score is the harmonic mean of precision and recall, providing a single metric that balances both. It is calculated as
  (20) $$f_{m}=2\cdot \frac{\mathrm{Precision}\;\mathrm{Macro}\cdot \mathrm{Recall}\;\mathrm{Macro}}{\mathrm{Precision}\;\mathrm{Macro}+\mathrm{Recall}\;\mathrm{Macro}}$$
- **Recall Macro (r_m)**: Recall macro is the average recall score over all classes, considering class imbalance by treating all classes equally. It is defined as
  (21) $$r_{m}=\frac{1}{n}\sum_{i=1}^{n}\frac{TP_{i}}{TP_{i}+FN_{i}}$$
  where $TP_{i}$ and $FN_{i}$ represent the true positives and false negatives for each class *i*, and *n* is the number of classes.
- **Precision Macro (p_m)**: Precision macro is the average precision score over all classes. It is defined as
  (22) $$p_{m}=\frac{1}{n}\sum_{i=1}^{n}\frac{TP_{i}}{TP_{i}+FP_{i}}$$
  where $TP_{i}$ and $FP_{i}$ are the true positives and false positives for each class *i*, and *n* is the number of classes.

#### 6.2.2. Class-Specific Metrics

In imbalanced classification problems, it is important to evaluate the performance on both the minority and majority classes separately. The following metrics are calculated for each class:

##### Minority Class Metrics

- **F-score minority (f_min)**: The F-score for the minority class, calculated similarly to the macro F-score, focuses specifically on the performance of the minority class:
  (23) $$f_{\min}=2\cdot \frac{\mathrm{Precision}\;\mathrm{Minority}\cdot \mathrm{Recall}\;\mathrm{Minority}}{\mathrm{Precision}\;\mathrm{Minority}+\mathrm{Recall}\;\mathrm{Minority}}$$
- **Recall minority (r_min)**: Recall for the minority class measures how well the minority class is identified:
  (24) $$r_{\min}=\frac{TP_{\min}}{TP_{\min}+FN_{\min}}$$
- **Precision Minority (p_min)**: Precision for the minority class measures the accuracy of positive predictions for the minority class:
  (25) $$p_{\min}=\frac{TP_{\min}}{TP_{\min}+FP_{\min}}$$

##### Majority Class Metrics

- **F-score majority (f1_may)**: The F-score for the majority class, calculated similarly to the macro F-score, focuses on the majority class:
  (26) $$f_{\mathrm{may}}=2\cdot \frac{\mathrm{Precision}\;\mathrm{Majority}\cdot \mathrm{Recall}\;\mathrm{Majority}}{\mathrm{Precision}\;\mathrm{Majority}+\mathrm{Recall}\;\mathrm{Majority}}$$
- **Recall majority (r_may)**: Recall for the majority class measures how well the majority class is identified:
  (27) $$r_{\mathrm{may}}=\frac{TP_{\mathrm{may}}}{TP_{\mathrm{may}}+FN_{\mathrm{may}}}$$
- **Precision majority (p_may)**: Precision for the majority class measures the accuracy of positive predictions for the majority class:
  (28) $$p_{\mathrm{may}}=\frac{TP_{\mathrm{may}}}{TP_{\mathrm{may}}+FP_{\mathrm{may}}}$$

#### 6.2.3. Total Features Selected (TFS)

Let TFS represent the number of selected features, which is defined as

(29) $$TFS=\sum_{i=1}^{m}I\left(f_{i}\right)$$

where *m* is the total number of features, and $I(f_{i})$ is an indicator function such that

$$I\left(f_{i}\right)=\left\{\begin{array}{cc} 1 & \mathrm{if}\;\mathrm{feature}\;f_{i}\;\mathrm{is}\;\mathrm{selected}\\ 0 & \mathrm{otherwise} \end{array}\right.$$

### 6.3. Experiment Results

The following tables present the results obtained from the experiments using the different classifiers (KNN, Random Forest, XGBoost) and objective functions. The values reported in each table represent the average and standard deviation for the defined evaluation metrics across 31 runs.

**Figure 9:**
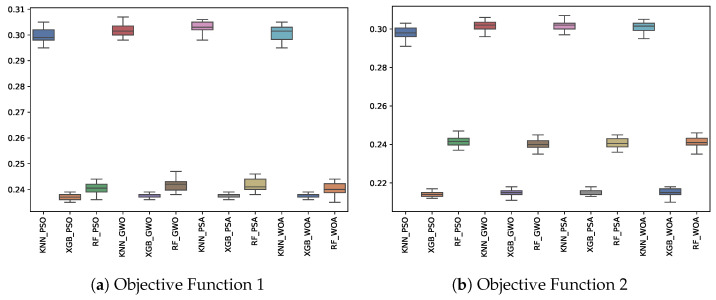
Fitness boxplots for each metaheuristic, classifier, and objective function used.

Table 7 shows the experimental results related to the fitness obtained. The table shows the fitness obtained by both objective functions (OF 1 and OF 2) for each metaheuristic with its corresponding classifier. The best fitness obtained, the average, and the standard deviation of the 31 independent runs are presented. The best results for each objective function are highlighted in bold. On the other hand, Figure 9 shows the fitness distribution for each experiment using a box–whisker plot. The x-axis corresponds to the metaheuristic–classifier pair, and the y-axis corresponds to the fitness.

From Table 7 and Figure 9, it can be noted that PSO paired with XGBoost produces the best overall results in minimizing the fitness function for both objective functions. This suggests that PSO is the most effective metaheuristic for this task, with XGBoost being the best classifier for achieving lower fitness values. While the other metaheuristics (GWO, PSA, WOA) also perform reasonably well, they are outperformed by PSO, particularly when paired with XGBoost. Therefore, the combination of PSO and XGBoost is recommended for minimizing both objective functions in this problem.

**Figure 10:**
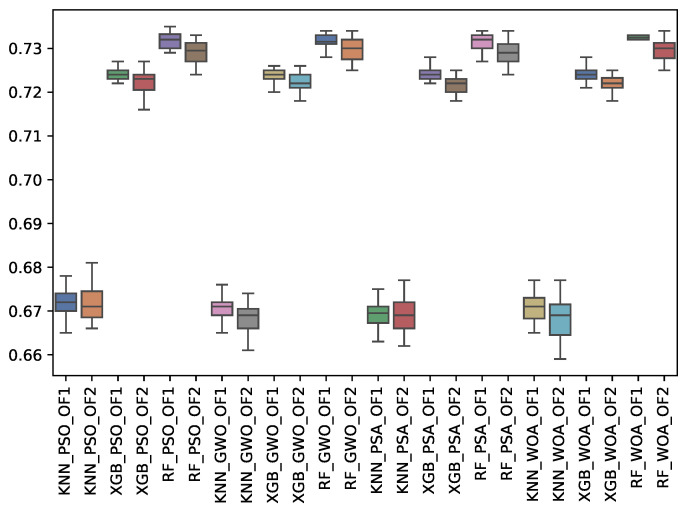
Boxplots of f1_m for each metaheuristic, classifier, and objective function used.

Table 8 shows the experimental results related to the F1-macro (f1_m) obtained. The table shows the f1_m obtained by both objective functions (OF 1 and OF 2) for each metaheuristic with its corresponding classifier. The average and the standard deviation of the 31 independent runs are presented. The best average for each objective function is highlighted in bold. On the other hand, Figure 10 shows the f1_m distribution for each experiment using a box-whisker plot. The x-axis corresponds to the metaheuristic–classifier pair, and the y-axis corresponds to the f1_m.

Table 8 and Figure 10 show the f1_m results across the classifiers and metaheuristics. The Random Forest (RF) consistently achieved the highest F1-macro score under Objective Functions 1 and 2 (OF 1 and OF 2), averaging 0.732 across the GWO, PSA, PSO, and WOA metaheuristics. K-Nearest Neighbors (KNN) displayed the lowest performance, with average values around 0.671 to 0.672, highlighting that RF is the most effective classifier in terms of overall macro performance. At the same time, KNN struggles with feature selection methods. The standard deviation across all results remains small, indicating stable performance.

**Figure 11:**
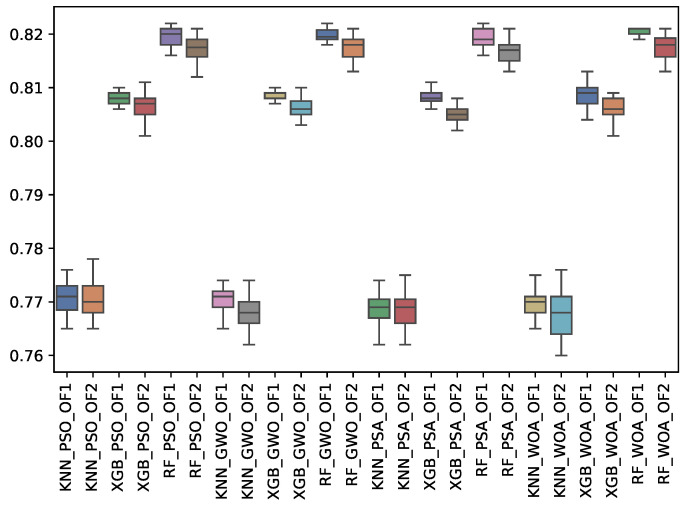
Boxplots of f1_may for each metaheuristic, classifier, and objective function used.

Table 9 shows the experimental results related to the F1-majority class (f1_may) obtained. The table shows the f1_may obtained by both objective functions (OF 1 and OF 2) for each metaheuristic with its corresponding classifier. The average and standard deviation of the 31 independent runs are presented. The best average for each objective function is highlighted in bold. On the other hand, Figure 11 shows the f1_may distribution for each experiment using a box–whisker plot. The x-axis corresponds to the metaheuristic–classifier pair, and the y-axis corresponds to the f1_may.

Like the F1-macro results, RF performs the best, achieving a peak F1-majority score of 0.820 for GWO and WOA under OF 1. XGBoost (XGB) performed slightly lower, with a peak of 0.808, while KNN remains the least effective.

**Figure 12:**
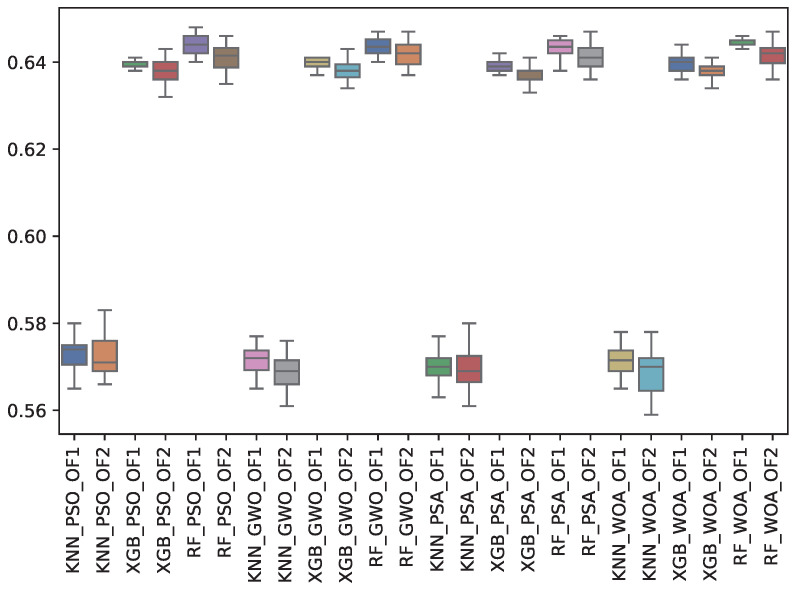
Boxplots of f1_min for each metaheuristic, classifier, and objective function used.

Table 10 shows the experimental results related to the F1-minority class (f1_min) obtained. The table shows the f1_min obtained by both objective functions (OF 1 and OF 2) for each metaheuristic with its corresponding classifier. The average and the standard deviation of the 31 independent runs are presented. The best average for each objective function is highlighted in bold. On the other hand, Figure 12 shows the f1_min distribution for each experiment using a box–whisker plot. The x-axis corresponds to the metaheuristic–classifier pair, and the y-axis corresponds to the f1_min.

The highest score was achieved by WOA, with an F1-minority score of 0.645 under RF. The other metaheuristics (GWO, PSA, PSO) also performed well with RF, indicating that feature selection by metaheuristics plays an important role in identifying minority class instances. KNN consistently showed the lowest performance in both objective functions.

**Figure 13:**
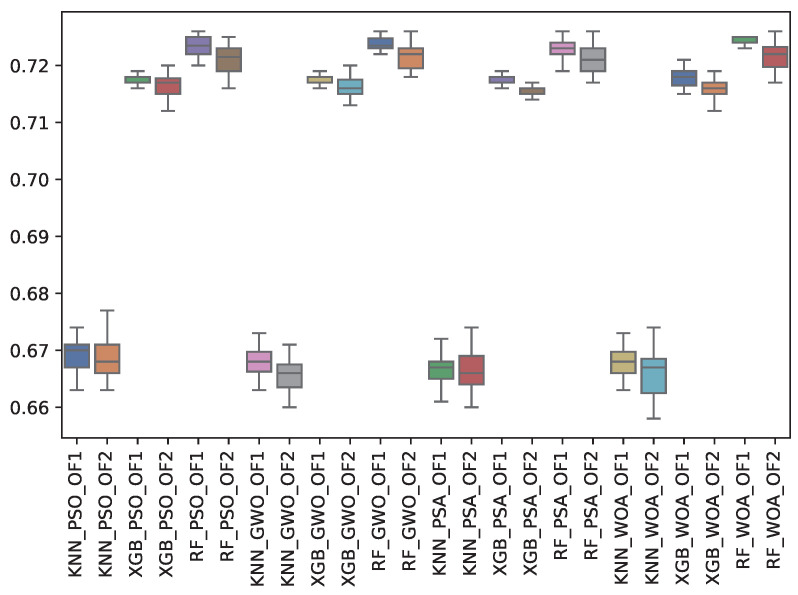
Boxplots of p_m for each metaheuristic, classifier, and objective function used.

**Figure 14:**
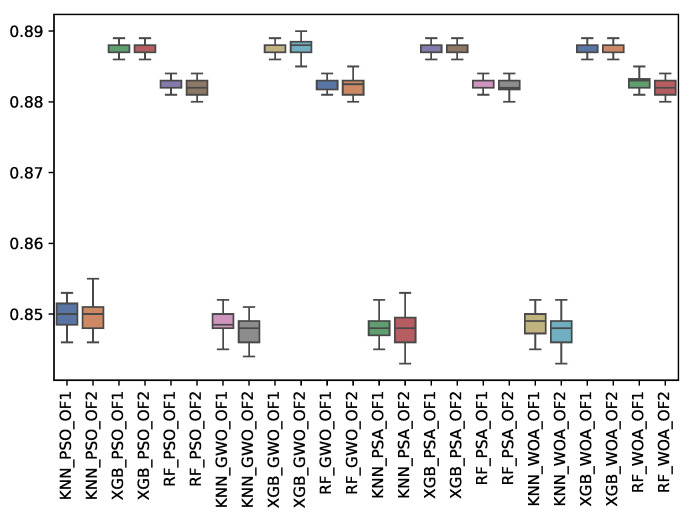
Boxplots of p_may for each metaheuristic, classifier, and objective function used.

**Figure 15:**
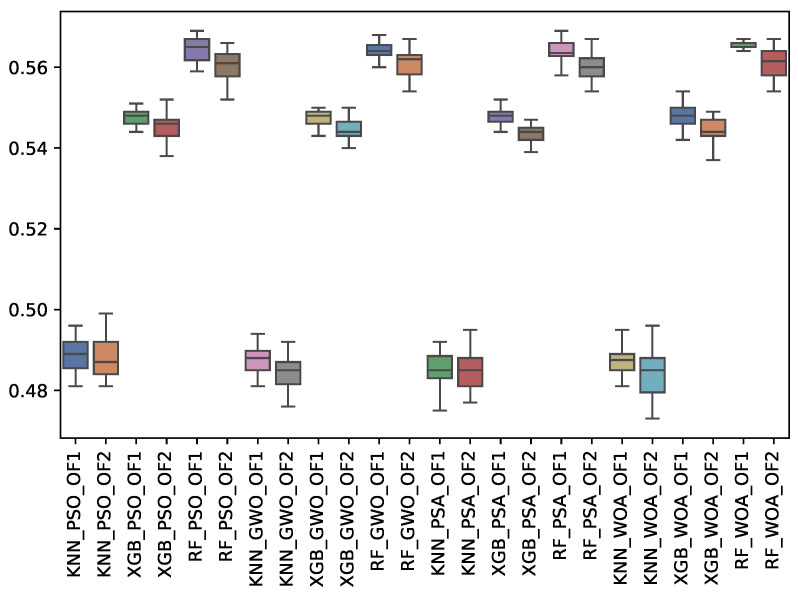
Boxplots of p_min for each metaheuristic, classifier, and objective function used.

Table 11, Table 12 and Table 13 show the experimental results related to the precision macro (p_m), majority (p_may), and minority classes (p_min) obtained. The tables show the p_m, p_may, and p_min obtained by both objective functions (OF 1 and OF 2) for each metaheuristic with its corresponding classifier. The average and standard deviation of the 31 independent runs are presented. The best average for each objective function is highlighted in bold. On the other hand, Figure 13, Figure 14 and Figure 15 show the p_m, p_may, and p_min distributions for each experiment using a box–whisker plot. The x-axis corresponds to the metaheuristic–classifier pair, and the y-axis corresponds to the p_m, p_may, and p_min.

For macro precision (p_m), RF achieved the highest value of 0.724 across GWO, PSA, PSO, and WOA metaheuristics. Regarding majority class precision (p_may), RF and XGB reached 0.888, while KNN showed slightly lower performance. Regarding minority class precision (p_min), WOA led with a score of 0.566, followed closely by PSO and GWO.

**Figure 16:**
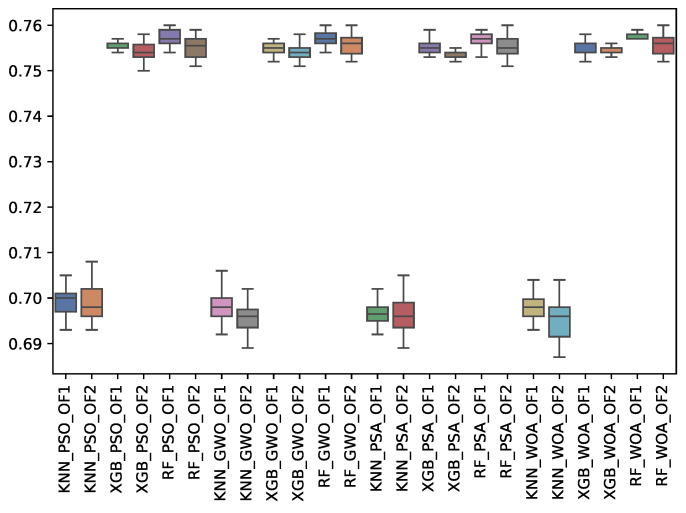
Boxplots of r_m for each metaheuristic, classifier, and objective function used.

**Figure 17:**
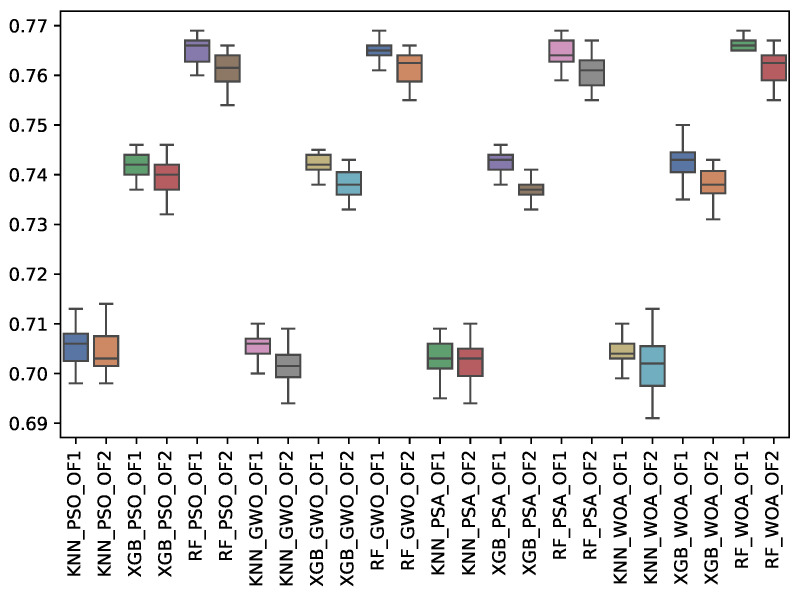
Boxplots of r_may for each metaheuristic, classifier, and objective function used.

**Figure 18:**
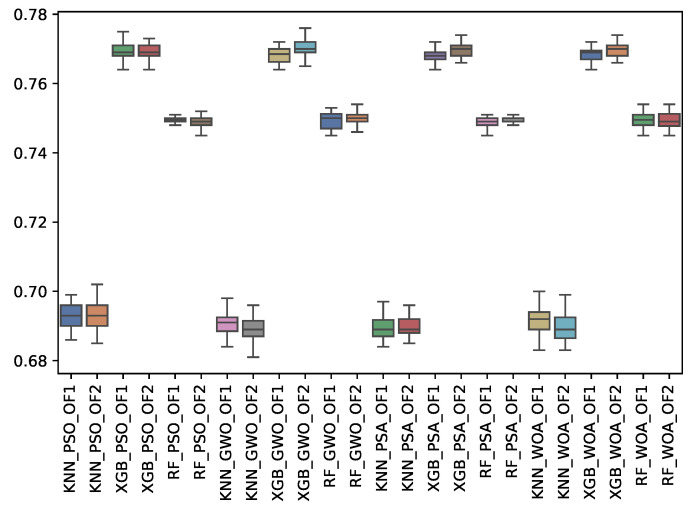
Boxplots of r_min for each metaheuristic, classifier, and objective function used.

Table 14, Table 15 and Table 16 show the experimental results related to the recall macro (r_m), majority (r_may), and minority classes (r_min) obtained. The tables show the r_m, r_may, and r_min obtained by both objective functions (OF 1 and OF 2) for each metaheuristic with its corresponding classifier. The average and the standard deviation of the 31 independent runs are presented. The best average for each objective function is highlighted in bold. On the other hand, Figure 16, Figure 17 and Figure 18 show the r_m, r_may, and r_min distributions for each experiment using a box–whisker plot. The x-axis corresponds to the metaheuristic–classifier pair, and the y-axis corresponds to the r_m, r_may, and r_min.

WOA achieved the highest recall for the minority class, with 0.770, while PSA and GWO performed well. For macro recall (r_m), RF scored 0.758, outperforming the other classifiers. In the majority class recall (r_may), WOA again took the lead, indicating its strong performance across multiple metrics.

**Figure 19:**
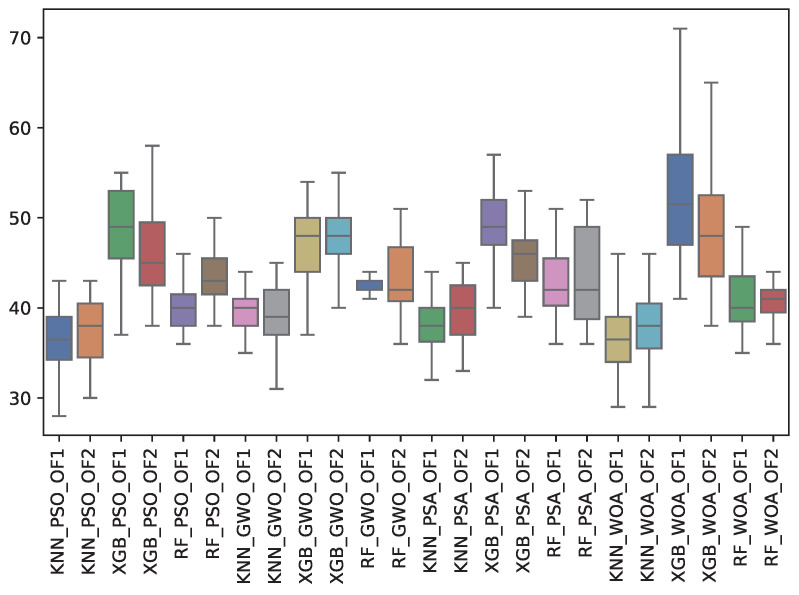
Boxplots of TFS for each metaheuristic, classifier, and objective function used.

Finally, Table 17 shows the total features selected (TFS) across the metaheuristics. The table shows the TFS obtained by both objective functions (OF 1 and OF 2) for each metaheuristic with its corresponding classifier. The average and the standard deviation of the 31 independent runs are presented. The best average for each objective function is highlighted in bold. On the other hand, Figure 19 shows the TFS distribution for each experiment using a box–whisker plot. The x-axis corresponds to the metaheuristic–classifier pair, and the y-axis corresponds to the TFS.

PSO demonstrated the most efficient feature selection, selecting the fewest features on average (36.5 for OF 1), while WOA selected the most features, reaching an average of 53.4 for XGB under OF 1. This suggests that while PSO is more aggressive in feature reduction, WOA maintains a more comprehensive feature set, which may contribute to its superior performance in minority class identification.

Overall, Random Forest emerged as the top-performing classifier across most metrics, particularly in the F1-macro, precision, and recall scores for both the majority and minority classes. WOA and PSO stood out as the most effective metaheuristics, with WOA excelling in minority class recall and PSO showing the best feature reduction capabilities. KNN consistently underperformed, suggesting it may not be suitable for this specific feature selection problem.

### 6.4. Statistical Test

A robust way to compare different algorithms is through statistical testing [62,76]. Our data does not follow a normal distribution, so we must perform a nonparametric test. Given this, we have applied the following comparative methodology:

- Apply the Friedman test to determine if there is an overall statistical difference between all the algorithms.
- If the Friedman test is positive (*p*-value < 0.05), the Neminyi post hoc test is applied to identify the pairs of algorithms with statistical differences.
- Once the pairs have a statistical difference, the Wilcoxon signed-rank test will be applied to determine the directionality of the statistical difference, that is, to determine which algorithm is better than the other.

Table 18 shows the *p*-value obtained after applying the Friedman test for all experiments carried out considering Objective Functions 1 and 2. As can be seen, in both objective functions, a *p*-value lower than 0.05 is obtained; therefore, there is a global statistical difference, and we can apply the Neminyi post hoc test to determine the pairs with significant differences.

Table 19 and Table 20 show the *p*-values obtained after applying the Neminyi post hoc test considering the fitness of Objective Function 1 and Objective Function 2, respectively. Analyzing these tables, we observe the following:

- From Table 19, 34 pairs with significant differences are observed.
- From Table 20, 38 pairs with significant differences are observed.

The Wilcoxon signed-rank test was performed because the data did not follow a normal distribution, and each experiment was conducted independently. We apply this test to determine the directionality of the statistical difference detected between these pairs in the previous step. Furthermore, we considered a significance level of 0.05, where the hypotheses are as follows:

(30) $$\begin{array}{c} H_{0}=Optimizer_{A}\geq Optimizer_{B}\\ H_{1}=Optimizer_{A}<Optimizer_{B} \end{array}$$

If the result of the statistical test is a *p*-value <0.05, it cannot be assumed that $Optimizer_{A}$ performs less well than $Optimizer_{B}$, and rejects $H_{0}$. This comparison is performed because our problem is a minimization problem.

We have used the *scipy.stats.wilcoxon* function from the Python scipy library. Among the configuration parameters for this function is the “alternative” parameter, which we defined with the value “less.” With this configuration, the applied test tells us whether $Optimizer_{A}$ is statistically less than $Optimizer_{B}$, in other words, whether $Optimizer_{A}$ is better than $Optimizer_{B}$, since we are working on a minimization problem.

Table 21 shows the number of times each algorithm was statistically better than another after applying the statistical test. The *p*-values for the statistical test are shown in Table 22 and Table 23. Table 21 shows that the algorithms using the KNN classifier are not statistically better than any algorithm for both objective functions. On the other hand, the algorithms incorporating the RF classifier are shown to be statistically better than other algorithms for both objective functions. A review of Table 22 and Table 23 shows that these triumphs are over algorithms that incorporate KNN. Finally, the best algorithms incorporate XGB as a classifier for both objective functions. Specifically, these algorithms are statistically better than ones that incorporate KNN and others that incorporate RF.

The algorithm that stands out the most is XGB_PSO, which triumphs 6 times in Objective Function 1 and 8 times in Objective Function 2. It is followed by the XGB_WOA algorithm, which triumphs 6 times in Objective Function 1 and 7 times in Objective Function 2. Closing the top 3, we find the XGB_PSA algorithm, which triumphs only 4 times for Objective Function 1 but 7 times for Objective Function 2.

## 7. Conclusions

This study demonstrated that employing metaheuristic techniques significantly reduced the features required to train machine learning models for predicting intradialytic hypotension (IDH) in dialysis patients. The XGBoost model, in particular, outperformed other classifiers such as Random Forest and K-Nearest Neighbors. Combined with feature selection techniques optimized by Particle Swarm Optimization (PSO), the XGBoost model achieved higher performance in minority class recall, minimizing false positives for hypotensive patients.

As mentioned in Section 1, our research hypothesis indicates that it is possible to detect an optimal combination of clinical and analytical parameters associated with the development of hypotension during dialysis. The results confirmed this hypothesis, as the optimized models could accurately predict hypotensive episodes with reduced features.

On the other hand, the main objective of improving the accuracy of IDH prediction while minimizing the number of required features was successfully achieved. The results of the study highlight the practical application of metaheuristics in healthcare, particularly in optimizing predictive models for critical conditions such as IDH.

These findings improve patient care by enabling early detection of hypotensive episodes, allowing for enhanced optimization of medical resources during dialysis sessions. In particular, it is observed that the XGBoost classifier outperforms both Random Forest and KNN. Furthermore, the incorporation of PSO into XGBoost delivers better experimental results than the rest, being statistically validated with tests.

While the results of this study demonstrate the effectiveness of metaheuristics in optimizing predictive models for intradialytic hypotension (IDH), several avenues for future research are worth exploring:

- Algorithm optimization beyond feature selection: In addition to feature selection, future studies could explore the optimization of hyperparameters of machine learning models.
- Application of deep learning models: Investigating the application of deep learning models, such as recurrent neural networks (RNNs) or long short-term memory networks (LSTMs), may provide further insight into complex relationships in the data and improve predictive accuracy.
- Personalized prediction models: Future research could focus on creating personalized prediction models based on individual patient profiles and climatic or geographic characteristics.
- Clinical validation and implementation: Validation through real-world clinical trials is necessary to bring the predictive model into clinical practice. This would involve integrating the model into dialysis machines or clinical decision support systems to assess its efficacy in a live healthcare setting. This gives rise to another future research oriented towards Explainable Artificial Intelligence. With this, models seek to be interpretable by professionals outside the field, such as medical professionals.

These future directions provide a framework to further the findings of this study, advance the role of predictive analytics in nephrology, and improve patient care through the integration of artificial intelligence.

## Figures and Tables

**Table 1:** IDH dataset.

		Labels		Features	
	Instances	Non Hypotensive	Hypotensive	Pre.	Post.
IDH dataset	68,574	48,764 (71.11%)	19,810 (28.89%)	71	87

**Table 2:** Classification results without optimization of the prediction model.

Classifier	f1_m	r_m	p_m	fs_min	r_min	p_min	fs_may	r_may	p_may
XGBoost	0.737	0.721	0.769	0.607	0.529	0.711	0.867	0.913	0.827
RF	0.717	0.698	0.763	0.570	0.474	0.715	0.864	0.923	0.812
KNN	0.651	0.641	0.677	0.474	0.405	0.571	0.828	0.876	0.784

**Table 3:** S-shaped and V-shaped transfer functions.

S-Shaped		V-Shaped	
Name	Equation	Name	Equation
S1	$T\left(d_{i}^{j}\right)=\frac{1}{1+e^{-2d_{i}^{j}}}$	V1	$T\left(d_{i}^{j}\right)=\left|erf\left(\frac{\sqrt{\pi }}{2}d_{i}^{j}\right)\right|$
S2	$T\left(d_{i}^{j}\right)=\frac{1}{1+e^{-d_{i}^{j}}}$	V2	$T\left(d_{i}^{j}\right)=\left|tanh(d_{i}^{j})\right|$
S3	$T\left(d_{i}^{j}\right)=\frac{1}{1+e^{\frac{-d_{i}^{j}}{2}}}$	V3	$T\left(d_{i}^{j}\right)=\left|\frac{d_{i}^{j}}{\sqrt{1+{\left(d_{i}^{j}\right)}^{2}}}\right|$
S4	$T\left(d_{i}^{j}\right)=\frac{1}{1+e^{\frac{-d_{i}^{j}}{3}}}$	V4	$T\left(d_{i}^{j}\right)=\left|\frac{2}{\pi }arctan\left(\frac{\pi }{2}d_{i}^{j}\right)\right|$

**Table 4:** Binarization rules.

Type	Binarization Rules
Standard	$X_{\mathrm{new}}^{j}=\left\{\begin{array}{cc} 1 & \mathrm{if}\;\mathrm{rand}\leq T(d_{i}^{j})\\ 0 & \mathrm{else}. \end{array}\right.$
Complement	$X_{\mathrm{new}}^{j}=\left\{\begin{array}{cc} \mathrm{Complement}(X_{w}^{j}) & \mathrm{if}\;\mathrm{rand}\leq T(d_{i}^{j})\\ 0 & \mathrm{else}. \end{array}\right.$
Static Probability	$X_{new}^{j}=\left\{\begin{array}{cc} 0 & if\;T(d_{i}^{j})\leq \alpha \\ X_{w}^{j} & if\;\alpha <T\left(d_{i}^{j}\right)\leq \frac{1}{2}\left(1+\alpha \right)\\ 1 & if\;T\left(d_{i}^{j}\right)\geq \frac{1}{2}\left(1+\alpha \right) \end{array}\right.$
Elitist	$X_{new}^{j}=\left\{\begin{array}{cc} X_{Best}^{j} & if\;rand<T(d_{i}^{j})\\ 0 & else. \end{array}\right.$
Roulette Elitist	$X_{new}^{j}=\left\{\begin{array}{cc} P\left[X_{new}^{j}=\zeta_{j}\right]=\frac{f(\zeta )}{\sum_{\delta \in Q_{g}}f\left(\delta \right)} & \mathrm{if}\;\mathrm{rand}\;\leq T(d_{i}^{j})\\ P[X_{new}^{j}=0]=1 & else. \end{array}\right.$

**Table 5:** Results of classifiers with RandomUnderSampler and varying parameters.

Classifier	Parameter	f1_m	r_m	p_m	f1_min	r_min	p_min	f1_may	r_may	p_may
KNN	1.0	0.632	0.659	0.634	0.527	0.652	0.443	0.737	0.666	0.825
Random Forest	1.0	0.72	0.746	0.712	0.629	0.738	0.549	0.81	0.753	0.876
XGBoost	1.0	0.725	0.756	0.718	0.64	0.766	0.549	0.81	0.745	0.887
KNN	0.9	0.64	0.66	0.638	0.526	0.622	0.455	0.754	0.698	0.82
Random Forest	0.9	0.727	0.745	0.718	0.631	0.709	0.568	0.822	0.781	0.869
XGBoost	0.9	0.734	0.758	0.725	0.645	0.745	0.569	0.823	0.771	0.881
KNN	0.8	0.646	0.66	0.642	0.523	0.59	0.47	0.769	0.729	0.814
Random Forest	0.8	0.732	0.743	0.725	0.63	0.677	0.59	0.834	0.809	0.86
XGBoost	0.8	0.739	0.756	0.731	0.646	0.718	0.587	0.833	0.795	0.874
KNN	0.7	0.652	0.659	0.648	0.519	0.553	0.488	0.786	0.765	0.808
Random Forest	0.7	0.734	0.737	0.732	0.625	0.636	0.613	0.843	0.837	0.85
XGBoost	0.7	0.744	0.753	0.737	0.645	0.686	0.609	0.843	0.821	0.866
KNN	0.6	0.655	0.655	0.655	0.509	0.508	0.509	0.8	0.801	0.8
Random Forest	0.6	0.736	0.73	0.743	0.618	0.593	0.646	0.854	0.868	0.84
XGBoost	0.6	0.745	0.745	0.745	0.638	0.64	0.636	0.852	0.851	0.853

**Table 6:** Setting parameters for each MH.

MH	Parameter	Value
PSO	$V_{Max}$	5000
	$c_{1}$	2
	$c_{2}$	2
	$w_{max}$	0.9
	$w_{min}$	0.2
GWO	a	decreases linearly from 2 to 0
WOA	a	decreases linearly from 2 to 0
	b	1
PSA	free parameters	

**Table 7:** Results by evaluation metric: fitness (best, average, and standard deviation).

	OF 1									OF 2								
	KNN			RF			XGB			KNN			RF			XGB		
Fitness	Best	Avg.	Std.	Best	Avg.	Std.	Best	Avg.	Std.	Best	Avg.	Std.	Best	Avg.	Std.	Best	Avg.	Std.
GWO	0.298	0.302	0.003	0.238	0.242	0.002	0.236	0.238	0.001	0.296	0.302	0.003	0.235	0.240	0.003	0.211	0.215	0.002
PSA	0.298	0.303	0.002	0.238	0.242	0.002	0.236	0.238	0.001	0.297	0.302	0.003	0.236	0.241	0.003	0.213	0.215	0.001
PSO	0.295	0.299	0.003	0.236	0.240	0.002	**0.235**	**0.237**	**0.001**	0.291	0.298	0.003	0.237	0.242	0.002	**0.212**	**0.214**	**0.001**
WOA	0.295	0.301	0.003	0.235	0.240	0.002	0.236	0.238	0.001	0.295	0.301	0.003	0.235	0.241	0.003	0.210	0.215	0.002

**Table 8:** Results by evaluation metric: f1_m (average and standard deviation).

	OF 1						OF 2					
	KNN		RF		XGB		KNN		RF		XGB	
f1_m	Avg.	Std.	Avg.	Std.	Avg.	Std.	Avg.	Std.	Avg.	Std.	Avg.	Std.
GWO	0.671	0.002	**0.732**	0.002	0.724	0.001	0.668	0.004	0.730	0.003	0.722	0.002
PSA	0.669	0.003	0.731	0.002	0.724	0.001	0.669	0.004	0.729	0.002	0.721	0.002
PSO	0.672	0.003	**0.732**	0.002	0.724	0.001	0.671	0.004	0.729	0.003	0.722	0.003
WOA	0.671	0.003	**0.732**	0.001	0.724	0.002	0.668	0.004	0.729	0.003	0.722	0.002

**Table 9:** Results by evaluation metric: f1_may (average and standard deviation).

	OF 1						OF 2					
	KNN		RF		XGB		KNN		RF		XGB	
f1_may	Avg.	Std.	Avg.	Std.	Avg.	Std.	Avg.	Std.	Avg.	Std.	Avg.	Std.
GWO	0.770	0.002	**0.820**	0.001	0.809	0.001	0.768	0.003	0.817	0.002	0.806	0.002
PSA	0.769	0.003	0.819	0.002	0.808	0.001	0.768	0.003	0.817	0.002	0.805	0.002
PSO	0.771	0.003	**0.820**	0.002	0.808	0.001	0.770	0.003	0.817	0.002	0.806	0.002
WOA	0.770	0.002	**0.820**	0.001	0.808	0.002	0.768	0.004	0.818	0.002	0.806	0.002

**Table 10:** Results by evaluation metric: f1_min (average and standard deviation).

	OF 1						OF 2					
	KNN		RF		XGB		KNN		RF		XGB	
f1_min	Avg.	Std.	Avg.	Std.	Avg.	Std.	Avg.	Std.	Avg.	Std.	Avg.	Std.
GWO	0.572	0.003	0.644	0.002	0.640	0.001	0.569	0.004	0.642	0.003	0.638	0.002
PSA	0.570	0.003	0.643	0.002	0.639	0.001	0.569	0.004	0.641	0.003	0.637	0.002
PSO	0.573	0.004	0.644	0.002	0.640	0.001	0.573	0.004	0.641	0.003	0.638	0.003
WOA	0.572	0.003	**0.645**	0.001	0.640	0.002	0.569	0.005	0.642	0.003	0.638	0.002

**Table 11:** Results by evaluation metric: p_m (average and standard deviation).

	OF 1						OF 2					
	KNN		RF		XGB		KNN		RF		XGB	
p_m	Avg.	Std.	Avg.	Std.	Avg.	Std.	Avg.	Std.	Avg.	Std.	Avg.	Std.
GWO	0.668	0.002	**0.724**	0.001	0.718	0.001	0.666	0.003	0.721	0.002	0.716	0.002
PSA	0.667	0.003	0.723	0.002	0.717	0.001	0.666	0.003	0.721	0.002	0.716	0.001
PSO	0.669	0.003	**0.724**	0.002	0.718	0.001	0.669	0.003	0.721	0.002	0.716	0.002
WOA	0.668	0.003	**0.724**	0.001	0.718	0.002	0.666	0.004	0.722	0.003	0.716	0.002

**Table 12:** Results by evaluation metric: p_may (average and standard deviation).

	OF 1						OF 2					
	KNN		RF		XGB		KNN		RF		XGB	
p_may	Avg.	Std.	Avg.	Std.	Avg.	Std.	Avg.	Std.	Avg.	Std.	Avg.	Std.
GWO	0.849	0.002	0.882	0.001	0.887	0.001	0.847	0.002	0.882	0.001	**0.888**	0.001
PSA	0.848	0.002	0.882	0.001	0.887	0.001	0.848	0.002	0.882	0.001	0.887	0.001
PSO	0.850	0.002	0.883	0.001	**0.888**	0.001	0.850	0.002	0.882	0.001	**0.888**	0.001
WOA	0.849	0.002	0.883	0.001	0.887	0.001	0.848	0.002	0.882	0.001	**0.888**	0.001

**Table 13:** Results by evaluation metric: p_min (average and standard deviation).

	OF 1						OF 2					
	KNN		RF		XGB		KNN		RF		XGB	
p_min	Avg.	Std.	Avg.	Std.	Avg.	Std.	Avg.	Std.	Avg.	Std.	Avg.	Std.
GWO	0.488	0.003	0.564	0.002	0.547	0.002	0.484	0.004	0.561	0.004	0.545	0.003
PSA	0.485	0.004	0.564	0.003	0.548	0.002	0.485	0.005	0.560	0.003	0.543	0.002
PSO	0.489	0.004	0.565	0.003	0.548	0.002	0.488	0.005	0.560	0.004	0.545	0.003
WOA	0.487	0.003	**0.566**	0.001	0.548	0.003	0.484	0.005	0.561	0.004	0.544	0.003

**Table 14:** Results by evaluation metric: r_m (average and standard deviation).

	OF 1						OF 2					
	KNN		RF		XGB		KNN		RF		XGB	
r_m	Avg.	Std.	Avg.	Std.	Avg.	Std.	Avg.	Std.	Avg.	Std.	Avg.	Std.
GWO	0.698	0.003	0.757	0.002	0.755	0.001	0.696	0.003	0.756	0.002	0.754	0.002
PSA	0.697	0.003	0.757	0.002	0.755	0.001	0.696	0.004	0.755	0.002	0.754	0.001
PSO	0.699	0.003	0.757	0.002	0.755	0.001	0.699	0.004	0.755	0.002	0.754	0.002
WOA	0.698	0.003	**0.758**	0.001	0.755	0.001	0.696	0.004	0.756	0.002	0.754	0.001

**Table 15:** Results by evaluation metric: r_may (average and standard deviation).

	OF 1						OF 2					
	KNN		RF		XGB		KNN		RF		XGB	
r_may	Avg.	Std.	Avg.	Std.	Avg.	Std.	Avg.	Std.	Avg.	Std.	Avg.	Std.
GWO	0.706	0.002	0.765	0.002	0.742	0.002	0.702	0.004	0.761	0.003	0.738	0.003
PSA	0.703	0.004	0.765	0.003	0.742	0.002	0.702	0.004	0.761	0.003	0.737	0.002
PSO	0.706	0.004	0.765	0.003	0.742	0.002	0.705	0.004	0.761	0.004	0.739	0.003
WOA	0.704	0.002	**0.766**	0.001	0.742	0.003	0.701	0.005	0.762	0.003	0.738	0.003

**Table 16:** Results by evaluation metric: r_min (average and standard deviation).

	OF 1						OF 2					
	KNN		RF		XGB		KNN		RF		XGB	
r_min	Avg.	Std.	Avg.	Std.	Avg.	Std.	Avg.	Std.	Avg.	Std.	Avg.	Std.
GWO	0.691	0.003	0.749	0.002	0.768	0.002	0.689	0.003	0.750	0.002	**0.770**	0.002
PSA	0.690	0.003	0.749	0.002	0.768	0.002	0.690	0.003	0.749	0.001	**0.770**	0.002
PSO	0.693	0.004	0.749	0.001	0.769	0.002	0.693	0.004	0.749	0.002	0.769	0.002
WOA	0.691	0.004	0.750	0.002	0.768	0.002	0.689	0.004	0.749	0.002	**0.770**	0.002

**Table 17:** Results by evaluation metric: total features selected (TFS) (average and standard deviation).

	OF 1						OF 2					
	KNN		RF		XGB		KNN		RF		XGB	
TFS	Avg.	Std.	Avg.	Std.	Avg.	Std.	Avg.	Std.	Avg.	Std.	Avg.	Std.
GWO	39.4	2.2	42.6	0.8	46.8	4.2	39.6	3.3	43.4	4.2	48.1	3.6
PSA	38.2	2.8	42.6	4.6	49.2	4.1	39.3	3.5	43.6	5.6	45.7	3.7
PSO	**36.5**	3.4	39.9	2.7	48.5	4.8	37.5	3.7	43.6	3.5	46.0	4.5
WOA	37.0	4.2	41.0	3.9	53.4	7.7	37.9	3.9	40.4	2.4	48.7	6.1

**Table 18:** Friedman test for objective functions OF 1 and OF 2.

	OF1	OF2
*p*-value	$1.51\times 10^{-34}$	$5.09\times 10^{-35}$

**Table 19:** Neminyi post hoc test considering OF 1.

Algorithm	KNN_GWO	KNN_PSA	KNN_PSO	KNN_WOA	RF_GWO	RF_PSA	RF_PSO	RF_WOA	XGB_GWO	XGB_PSA	XGB_PSO	XGB_WOA
KNN_GWO	X	-	-	-	$4.69\times 10^{-02}$	$4.38\times 10^{-02}$	$1.53\times 10^{-03}$	$5.83\times 10^{-04}$	$7.04\times 10^{-10}$	$1.55\times 10^{-08}$	$3.81\times 10^{-10}$	$1.74\times 10^{-09}$
KNN_PSA	-	X	-	-	$2.43\times 10^{-03}$	$2.22\times 10^{-03}$	$3.30\times 10^{-05}$	$1.04\times 10^{-05}$	$1.83\times 10^{-12}$	$5.81\times 10^{-11}$	$9.25\times 10^{-13}$	$5.02\times 10^{-12}$
KNN_PSO	-	-	X	-	-	-	$1.23\times 10^{-02}$	$5.39\times 10^{-03}$	$2.36\times 10^{-08}$	$4.09\times 10^{-07}$	$1.34\times 10^{-08}$	$5.45\times 10^{-08}$
KNN_WOA	-	-	-	X	-	-	$3.48\times 10^{-03}$	$1.39\times 10^{-03}$	$2.71\times 10^{-09}$	$5.45\times 10^{-08}$	$1.50\times 10^{-09}$	$6.53\times 10^{-09}$
RF_GWO	$4.69\times 10^{-02}$	$2.43\times 10^{-03}$	-	-	X	-	-	-	$2.29\times 10^{-02}$	-	$1.69\times 10^{-02}$	$3.55\times 10^{-02}$
RF_PSA	$4.38\times 10^{-02}$	$2.22\times 10^{-03}$	-	-	-	X	-	-	$2.47\times 10^{-02}$	-	$1.82\times 10^{-02}$	$3.81\times 10^{-02}$
RF_PSO	$1.53\times 10^{-03}$	$3.30\times 10^{-05}$	$1.23\times 10^{-02}$	$3.48\times 10^{-03}$	-	-	X	-	-	-	-	-
RF_WOA	$5.83\times 10^{-04}$	$1.04\times 10^{-05}$	$5.39\times 10^{-03}$	$1.39\times 10^{-03}$	-	-	-	X	-	-	-	-
XGB_GWO	$7.04\times 10^{-10}$	$1.83\times 10^{-12}$	$2.36\times 10^{-08}$	$2.71\times 10^{-09}$	$2.29\times 10^{-02}$	$2.47\times 10^{-02}$	-	-	X	-	-	-
XGB_PSA	$1.55\times 10^{-08}$	$5.81\times 10^{-11}$	$4.09\times 10^{-07}$	$5.45\times 10^{-08}$	-	-	-	-	-	X	-	-
XGB_PSO	$3.81\times 10^{-10}$	$9.25\times 10^{-13}$	$1.34\times 10^{-08}$	$1.50\times 10^{-09}$	$1.69\times 10^{-02}$	$1.82\times 10^{-02}$	-	-	-	-	X	-
XGB_WOA	$1.74\times 10^{-09}$	$5.02\times 10^{-12}$	$5.45\times 10^{-08}$	$6.53\times 10^{-09}$	$3.55\times 10^{-02}$	$3.81\times 10^{-02}$	-	-	-	-	-	X

**Table 20:** Neminyi post hoc test considering OF 2.

Algorithm	KNN_GWO	KNN_PSA	KNN_PSO	KNN_WOA	RF_GWO	RF_PSA	RF_PSO	RF_WOA	XGB_GWO	XGB_PSA	XGB_PSO	XGB_WOA
KNN_GWO	X	-	-	-	$2.73\times 10^{-03}$	$1.02\times 10^{-02}$	$1.42\times 10^{-02}$	$1.81\times 10^{-02}$	$6.16\times 10^{-10}$	$1.07\times 10^{-10}$	$6.63\times 10^{-13}$	$9.09\times 10^{-11}$
KNN_PSA	-	X	-	-	$7.30\times 10^{-03}$	$2.46\times 10^{-02}$	$3.32\times 10^{-02}$	$4.13\times 10^{-02}$	$3.34\times 10^{-09}$	$6.16\times 10^{-10}$	$4.50\times 10^{-12}$	$5.27\times 10^{-10}$
KNN_PSO	-	-	X	-	-	-	-	-	$6.34\times 10^{-07}$	$1.43\times 10^{-07}$	$1.82\times 10^{-09}$	$1.25\times 10^{-07}$
KNN_WOA	-	-	-	X	$3.00\times 10^{-03}$	$1.11\times 10^{-02}$	$1.54\times 10^{-02}$	$1.95\times 10^{-02}$	$7.20\times 10^{-10}$	$1.26\times 10^{-10}$	$7.91\times 10^{-13}$	$1.07\times 10^{-10}$
RF_GWO	$2.73\times 10^{-03}$	$7.30\times 10^{-03}$	-	$3.00\times 10^{-03}$	X	-	-	-	-	-	$1.31\times 10^{-02}$	-
RF_PSA	$1.02\times 10^{-02}$	$2.46\times 10^{-02}$	-	$1.11\times 10^{-02}$	-	X	-	-	-	$4.13\times 10^{-02}$	$3.60\times 10^{-03}$	$3.84\times 10^{-02}$
RF_PSO	$1.42\times 10^{-02}$	$3.32\times 10^{-02}$	-	$1.54\times 10^{-02}$	-	-	X	-	-	$3.09\times 10^{-02}$	$2.49\times 10^{-03}$	$2.86\times 10^{-02}$
RF_WOA	$1.81\times 10^{-02}$	$4.13\times 10^{-02}$	-	$1.95\times 10^{-02}$	-	-	-	X	-	$2.46\times 10^{-02}$	$1.88\times 10^{-03}$	$2.28\times 10^{-02}$
XGB_GWO	$6.16\times 10^{-10}$	$3.34\times 10^{-09}$	$6.34\times 10^{-07}$	$7.20\times 10^{-10}$	-	-	-	-	X	-	-	-
XGB_PSA	$1.07\times 10^{-10}$	$6.16\times 10^{-10}$	$1.43\times 10^{-07}$	$1.26\times 10^{-10}$	-	$4.13\times 10^{-02}$	$3.09\times 10^{-02}$	$2.46\times 10^{-02}$	-	X	-	-
XGB_PSO	$6.63\times 10^{-13}$	$4.50\times 10^{-12}$	$1.82\times 10^{-09}$	$7.91\times 10^{-13}$	$1.31\times 10^{-02}$	$3.60\times 10^{-03}$	$2.49\times 10^{-03}$	$1.88\times 10^{-03}$	-	-	X	-
XGB_WOA	$9.09\times 10^{-11}$	$5.27\times 10^{-10}$	$1.25\times 10^{-07}$	$1.07\times 10^{-10}$	-	$3.84\times 10^{-02}$	$2.86\times 10^{-02}$	$2.28\times 10^{-02}$	-	-	-	X

**Table 21:** Summary Wilcoxon signed-rank test.

Algorithm	OF1	OF2
KNN_GWO	0	0
KNN_PSA	0	0
KNN_PSO	0	0
KNN_WOA	0	0
RF_GWO	2	3
RF_PSA	2	3
RF_PSO	4	3
RF_WOA	4	3
XGB_GWO	6	4
XGB_PSA	4	7
XGB_PSO	6	8
XGB_WOA	6	7

**Table 22:** Wilcoxon signed-rank test for pairs with significant difference from Table 19.

Comparison	*p*-Value	Conclusion
RF_GWO v/s KNN_GWO	0.0	RF_GWO is better than KNN_GWO
RF_GWO v/s KNN_PSA	0.0	RF_GWO is better than KNN_PSA
RF_PSA v/s KNN_GWO	0.0	RF_PSA is better than KNN_GWO
RF_PSA v/s KNN_PSA	0.0	RF_PSA is better than KNN_PSA
RF_PSO v/s KNN_GWO	0.0	RF_PSO is better than KNN_GWO
RF_PSO v/s KNN_PSA	0.0	RF_PSO is better than KNN_PSA
RF_PSO v/s KNN_PSO	0.0	RF_PSO is better than KNN_PSO
RF_PSO v/s KNN_WOA	0.0	RF_PSO is better than KNN_WOA
RF_WOA v/s KNN_GWO	0.0	RF_WOA is better than KNN_GWO
RF_WOA v/s KNN_PSA	0.0	RF_WOA is better than KNN_PSA
RF_WOA v/s KNN_PSO	0.0	RF_WOA is better than KNN_PSO
RF_WOA v/s KNN_WOA	0.0	RF_WOA is better than KNN_WOA
XGB_GWO v/s KNN_GWO	0.0	XGB_GWO is better than KNN_GWO
XGB_GWO v/s KNN_PSA	0.0	XGB_GWO is better than KNN_PSA
XGB_GWO v/s KNN_PSO	0.0	XGB_GWO is better than KNN_PSO
XGB_GWO v/s KNN_WOA	0.0	XGB_GWO is better than KNN_WOA
XGB_GWO v/s RF_GWO	0.0001	XGB_GWO is better than RF_GWO
XGB_GWO v/s RF_PSA	0.0	XGB_GWO is better than RF_PSA
XGB_PSA v/s KNN_GWO	0.0	XGB_PSA is better than KNN_GWO
XGB_PSA v/s KNN_PSA	0.0	XGB_PSA is better than KNN_PSA
XGB_PSA v/s KNN_PSO	0.0	XGB_PSA is better than KNN_PSO
XGB_PSA v/s KNN_WOA	0.0	XGB_PSA is better than KNN_WOA
XGB_PSO v/s KNN_GWO	0.0	XGB_PSO is better than KNN_GWO
XGB_PSO v/s KNN_PSA	0.0	XGB_PSO is better than KNN_PSA
XGB_PSO v/s KNN_PSO	0.0	XGB_PSO is better than KNN_PSO
XGB_PSO v/s KNN_WOA	0.0	XGB_PSO is better than KNN_WOA
XGB_PSO v/s RF_GWO	0.0001	XGB_PSO is better than RF_GWO
XGB_PSO v/s RF_PSA	0.0001	XGB_PSO is better than RF_PSA
XGB_WOA v/s KNN_GWO	0.0	XGB_WOA is better than KNN_GWO
XGB_WOA v/s KNN_PSA	0.0	XGB_WOA is better than KNN_PSA
XGB_WOA v/s KNN_PSO	0.0	XGB_WOA is better than KNN_PSO
XGB_WOA v/s KNN_WOA	0.0	XGB_WOA is better than KNN_WOA
XGB_WOA v/s RF_GWO	0.0001	XGB_WOA is better than RF_GWO
XGB_WOA v/s RF_PSA	0.0	XGB_WOA is better than RF_PSA

**Table 23:** Wilcoxon signed-rank test for pairs with significant difference from Table 20.

Comparison	*p*-Value	Conclusion
RF_GWO v/s KNN_GWO	0.0001	RF_GWO is better than KNN_GWO
RF_GWO v/s KNN_PSA	0.0001	RF_GWO is better than KNN_PSA
RF_GWO v/s KNN_WOA	0.0001	RF_GWO is better than KNN_WOA
RF_PSA v/s KNN_GWO	0.0001	RF_PSA is better than KNN_GWO
RF_PSA v/s KNN_PSA	0.0001	RF_PSA is better than KNN_PSA
RF_PSA v/s KNN_WOA	0.0001	RF_PSA is better than KNN_WOA
RF_PSO v/s KNN_GWO	0.0001	RF_PSO is better than KNN_GWO
RF_PSO v/s KNN_PSA	0.0001	RF_PSO is better than KNN_PSA
RF_PSO v/s KNN_WOA	0.0001	RF_PSO is better than KNN_WOA
RF_WOA v/s KNN_GWO	0.0001	RF_WOA is better than KNN_GWO
RF_WOA v/s KNN_PSA	0.0001	RF_WOA is better than KNN_PSA
RF_WOA v/s KNN_WOA	0.0001	RF_WOA is better than KNN_WOA
XGB_GWO v/s KNN_GWO	0.0001	XGB_GWO is better than KNN_GWO
XGB_GWO v/s KNN_PSA	0.0001	XGB_GWO is better than KNN_PSA
XGB_GWO v/s KNN_PSO	0.0001	XGB_GWO is better than KNN_PSO
XGB_GWO v/s KNN_WOA	0.0001	XGB_GWO is better than KNN_WOA
XGB_PSA v/s KNN_GWO	0.0001	XGB_PSA is better than KNN_GWO
XGB_PSA v/s KNN_PSA	0.0001	XGB_PSA is better than KNN_PSA
XGB_PSA v/s KNN_PSO	0.0001	XGB_PSA is better than KNN_PSO
XGB_PSA v/s KNN_WOA	0.0001	XGB_PSA is better than KNN_WOA
XGB_PSA v/s RF_PSA	0.0001	XGB_PSA is better than RF_PSA
XGB_PSA v/s RF_PSO	0.0001	XGB_PSA is better than RF_PSO
XGB_PSA v/s RF_WOA	0.0001	XGB_PSA is better than RF_WOA
XGB_PSO v/s KNN_GWO	0.0001	XGB_PSO is better than KNN_GWO
XGB_PSO v/s KNN_PSA	0.0001	XGB_PSO is better than KNN_PSA
XGB_PSO v/s KNN_PSO	0.0001	XGB_PSO is better than KNN_PSO
XGB_PSO v/s KNN_WOA	0.0001	XGB_PSO is better than KNN_WOA
XGB_PSO v/s RF_GWO	0.0001	XGB_PSO is better than RF_GWO
XGB_PSO v/s RF_PSA	0.0001	XGB_PSO is better than RF_PSA
XGB_PSO v/s RF_PSO	0.0001	XGB_PSO is better than RF_PSO
XGB_PSO v/s RF_WOA	0.0001	XGB_PSO is better than RF_WOA
XGB_WOA v/s KNN_GWO	0.0001	XGB_WOA is better than KNN_GWO
XGB_WOA v/s KNN_PSA	0.0001	XGB_WOA is better than KNN_PSA
XGB_WOA v/s KNN_PSO	0.0001	XGB_WOA is better than KNN_PSO
XGB_WOA v/s KNN_WOA	0.0001	XGB_WOA is better than KNN_WOA
XGB_WOA v/s RF_PSA	0.0001	XGB_WOA is better than RF_PSA
XGB_WOA v/s RF_PSO	0.0001	XGB_WOA is better than RF_PSO
XGB_WOA v/s RF_WOA	0.0001	XGB_WOA is better than RF_WOA

## Data Availability

In our study, the dataset was provided by the Instituto de Salud Carlos III, which conducted all initial data extraction, analysis, processing, and rigorous anonymization procedures. We utilized the resulting anonymized data for our research on predicting intradialytic hypotension, ensuring that no personally identifiable information was accessed or retained. This approach not only safeguards patient privacy but also upholds data transparency and reproducibility standards, as the provenance and processing of the data are clearly documented and independently verified.
